# Supplementary material for: Potential-dependent polaron formation activates TiO2 for the hydrogen evolution reaction
Source: Nat Commun. 2026 Jan 28;17:2104. doi: 10.1038/s41467-026-68892-5 (PMC12953770; doi:10.1038/s41467-026-68892-5)
Supplement: Supplementary file 1 — Supplementary Information [file 41467_2026_68892_MOESM1_ESM.pdf]

Supplementary Information

Potential-dependent polaron formation activates TiO_2 for the hydrogen evolution reaction

Tongwei Wu[1,*,‡], Xiaoxi Guo[5,‡], Guangjie Zhang[4], Yanning Zhang[1], Li Song[6], Zheng Liu[3,*], Hui Zhang[8], Shucheng Shi[7,*], Limin Liu[9], Marko M. Melander[2,*] and Karoliina Honkala[2,*]

[1]Institute of Fundamental and Frontier Sciences, University of Electronic Science and Technology of China, Chengdu, 610054 P. R. China

[2]Department of Chemistry, Nanoscience Center, University of Jyväskylä, Jyväskylä, FI-40014 Finland

[3]School of Materials Science and Engineering, Nanyang Technological University, Singapore, 639798 Singapore

[4]CAS Key Laboratory of Standardization and Measurement for Nanotechnology, National Center for Nanoscience and Technology, Beijing, 100190 P. R. China

[5]Precision Medicine Translational Research Center, West China Hospital, Sichuan University, Chengdu, 610041 P. R. China

[6]National Synchrotron Radiation Laboratory, CAS Center for Excellence in Nanoscience, University of Science and Technology of China, Hefei, 23002 P. R. China

[7]Center for Transformative Science, ShanghaiTech University, Shanghai, 201210 P. R. China

[8]Shanghai Synchrotron Radiation Facility, Shanghai Advanced Research Institute, Chinese Academy of Sciences, Shanghai, 201204 P. R. China

[9]School of Physics, Beihang University, Beijing, 100191 P. R. China

‡T. Wu and X. Guo contributed equally to this work.

Section S1. Computational and model details

The DFT calculations were carried out in the grid-based projector augmented wave (PAW) formalism as implemented in the GPAW 19. 8 .1 code.[1,2] The Kohn-Sham equations were solved on a uniform real-space grid with a 0.18 Å grid spacing. The exchange-correlation effects were accounted for by using the BEEF-vdW-functional, which combines the generalized gradient approximation with the Langreth-Lundqvist van der Waals-functional in an optimal way for accurate adsorption energies.[3] All calculations were performed spin-polarized. To reliably estimate the U values, we used the recent implementation[4] of self-consistent linear response method (LR-DFT+U) in GPAW software within the simplified, rotationally invariant DFT+U method by Dudarev et al. This implementation was used to compute the U value for the Ti d electrons and U_d (Ti) = 4.5 eV was obtained.

The solvent at the electrochemical interface was modeled using a hybrid of implicit/explicit approach[5] combining four-layer explicit solvent comprising 16 H_2O molecules in TiO_2(101) and two-layer explicit solvent comprising 10 H_2O molecules in Au (111), and a SCMVD dielectric continuum model for water[5] was exploited as implicit solvent to fill the rest. The atomic radii used by SCMVD to determine the cavity size were 2.2 Å for Ti and 1.5 Å for O. The positions and orientations of the explicit water molecules were optimized using the minima hopping global optimization method[6] as implemented in ASE.[7]

The theoretical lattice constants for Au (4.24 Å) and for TiO_2 (a = b = 3.836 Å and c = 9.841 Å) were used, which was found by minimizing the total energies of bulk Au and TiO_2. The computational lattice constants are slightly overestimated compared to experimental values of Au (4.08 Å)[8] and TiO_2 (a = b =3.782 Å and c = 9.502 Å)[9,10]. The TiO_2 (101) system was modelled as a 2 × 1 supercell with four (O-Ti-O) trilayers with the bottom trilayer fixed. The Au (111) surface was modelled as a 2 × 2 supercell with Au four layers with the bottom layer fixed. 25 Å of dielectric solvent was added on top of the slabs. The transition states (TSs) of each elementary reaction step were found using the constrained minimization approach,[11] and they were confirmed by the presence of a single imaginary vibrational mode along the reaction coordinate. A solvated H_3O^+ is considered as the initial state for the proton transfer reaction.

For the determination of Ti^{3+} polaron formation, while the primary role of DOS is to quantitatively describe the distribution of electronic energy levels, integrating the DOS over the given energy range ($\int_{E_1}^{E_2} \rho(\epsilon) d\epsilon$) provides the number of states between energies E_1 and E_2. However, this approach does not provide quantitative information about the spatial distribution of electrons. By correlating the atomic spin magnetic moment and DOS, it is possible to effectively capture both the spatial localization and the energy level of e.g. a polaronic state. This method is particularly applicable to non-magnetic TiO_2 systems where the quantitative relationship between magnetic moment and electron localization is clear. This is due to the single source of magnetic moment and the absence of spin coupling interference. The atomic magnetic moments in TiO_2 arise from excess electrons forming polarons so the DOS and atom spin magnetic moments together quantitatively describe electron localization and energy.

S1.1. Constant potential simulations

Computational methods within the grand canonical ensemble (GCE) have enabled explicit consideration of the electrode potential.[12-15] Most GCE-DFT methods achieve constant potential simulations by fixing the Fermi level or equivalently the work function of the simulation cell, but we observed that this approach is not suitable for the semiconductor electrodes, see Supplementary Section 1.1.1. Hence, in this work we have used the recent constant inner potential, CIP-DFT, which is a particular implementation of GCE-DFT[12]. The CIP-DFT method achieves constant potential calculations by iteratively adjusting the number of electrons in the simulation cell until the desired inner potential is obtained. The simulation cell is kept charge neutral by including a homogeneous counter charge within the implicit solvent regions of the unit cell. The electrode potential is defined as *inner potential* difference referenced to the inner potential at the potential of zero charge (PZC), where the whole charge on the electrode has been screened and no electric field is present.

The absolute electrode potential ($U^M(abs)$) computed from the electrode inner potential on the PZC scale is then determined as:

$$U^M(abs) = U^M(\sigma) - U^M(\sigma = 0) = -\mu_e^M[\sigma] - (-\mu_e^M[\sigma]) \quad (1)$$

$$= -\left(\mu_e^{0,M}[\sigma] - \phi^M(\sigma) + \phi^S(\sigma)\right) - \left(-\mu_e^{0,M}[\sigma = 0] + \phi^M(\sigma = 0) - \phi^S(\sigma = 0)\right)$$

$= \phi^M[\sigma] - \phi^M(\sigma = 0)$

where the electrode inner potentials, $\phi^M[\sigma]$, are referenced against the solution inner potential, ϕ^S which is set to zero using Dirichlet boundary conditions in the Poisson equation from which the electrostatic potential is solved.[12]

The absolute electrode potential vs the standard hydrogen electrode U_{SHE} is further defined as below:

$$U_{SHE} = U^M(abs) - U^{SHE}(abs) \quad (2)$$

where $U^{SHE}(abs)$ has been determined experimentally to be ~4.44 V.

The energy used in the analysis of electrode reactions is the grand free energy:

$$\Omega(\phi\colon N_e) = F(N) - (\mu^0 - \Delta\phi^{PZC})N_e \quad (3)$$

The reaction grand free energy (ΔG_r) of each elementary reaction was calculated as follow:

$$\Delta G_r = \Delta\Omega \quad (4)$$

The electrochemical rate constants at constant potential can be estimated within the grand canonical ensemble harmonic transition state theory[13] using grand free energies computed analogously to the reaction free energies.

We note that the calculation of acidic PCET process in this work was only possible via the use of this grand canonical framework, due to the potential-dependent H^+ transfer at semiconductor electrode-electrolyte interfaces.

The constant inner potential (CIP-DFT) molecular dynamics (MD) can be readily performed for the studies of the dynamics of explicitly solvated electrochemical interfaces as well as for moderately large systems investigated herein under constant (inner) potential conditions.[12] Hence, CIP-DFT-MD simulations of electrochemical systems can achieve fully consistent treatment of electrochemical interfaces and reactions. In this work, CIP-DFT-MD calculations were performed to confirm the dynamic mechanism of the potential-dependent Ti^{3+} polaron states on the TiO_2 (101) surface. Langevin dynamics (friction = 0.2 ps^{-1}) was used for the TiO_2 (101) system to control the temperature (set at 300 K) in the simulations with 1 fs time step and 2 u mass for hydrogen atoms, which was shown to provide a good control over the temperature and dynamics in our previous work[16]. The trajectories were computed with the following convergence criteria: eigenstates: $1.0e^{-4}$, density: $1.0e^{-5}$, energy: $1e^{-6}$ setting. Note

that the potential-dependent TiO_2 (101) surfaces were thermalised for 2 ps before the production runs.

S 1.1.1 Application of GCE-DFT and CIP-DFT semiconductor electrodes

While GCE-DFT can now be routinely applied to simulating metallic electrodes, the application of these methods to semiconductor surfaces has not been thoroughly validated. The main issue of the constant Fermi level GCE-DFT for semiconductors is the discontinuous electronic structure due to the presence of a bandgap, which means that the Fermi level and hence the electrode potential cannot be controlled continuously. In our recent work[12] we demonstrated that this discontinuity not only leads to poor numerical performance of GCE-DFT but also makes the electrode potential extremely sensitivity to surface charge: by changing the surface charge by $\pm 0.05e^-$ the TiO_2(101) electrode potential changes by ∓ 1.0 V as shown Supplementary Fig. 1, the constant E_F calculations. Similar sensitivity has also been observed before[17] and it has been shown that GCE-DFT calculations can become unreliable if the Fermi level crosses the bandgap during a reaction pathway.[18] In our present work this sensitivity between charge and electrode potential leads to computational results which are not in agreement with experiments. For instance, the Fermi levels and the corresponding electrode potentials at which electron polarons emerge are not consistent with experiments.

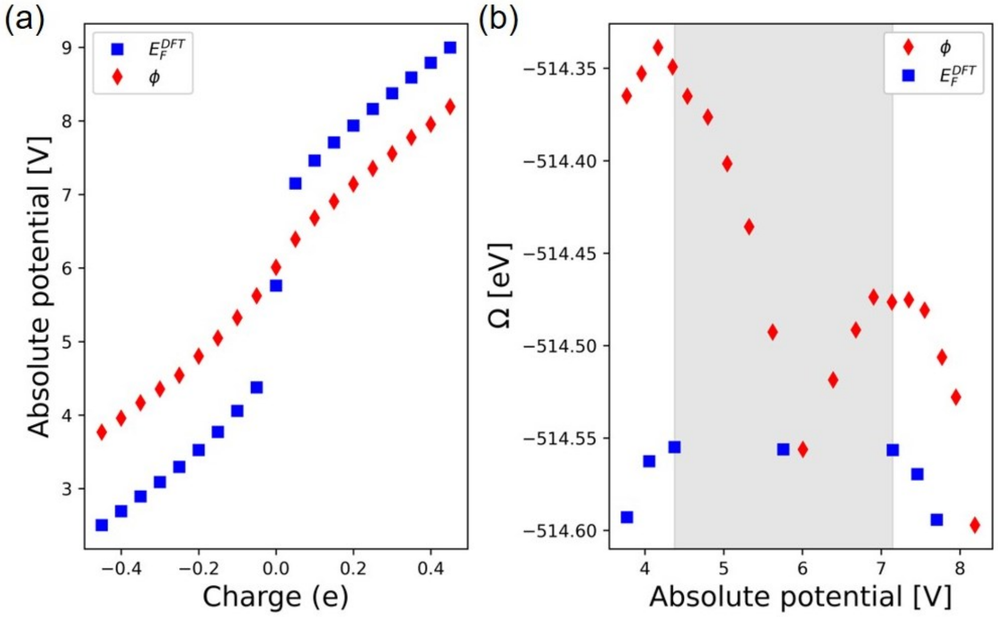

Supplementary Fig. 1 | Analysis of the TiO_2 (101) electrode. (a) Absolute potential as a function of charge. (b) The grand free energy as function of the absolute potential computed

using CIP-DFT, constant-E_F^{DFT} -DFT. The grey area indicates the band gap region of the pristine TiO_2(101) surface on the absolute potential scale.

Given these issues we applied the CIP-DFT method for semiconductors. As noted in our previous work, the CIP-DFT needs to be carefully benchmarked for semiconductors, in particular within the bandgap region. As shown in Supplementary Fig. 1, the CIP-DFT and constant Fermi level GCE-DFT calculations are clearly different within the bandgap; the surface charge (σ) and grand energy (Ω) from GCE-DFT are discontinuous while CIP-DFT exhibits a continuous behavior. However, the CIP-DFT grand energies are not convex within the bandgap region (grey region in Supplementary Fig. 1 b) which indicates electronic instability at these potentials and hence incorrect thermodynamics at the electrode potentials within the bandgap.[12] However, at the PZC and outside the bandgap both GCE-DFT exhibit similar trends for electronic capacitance $\left(C = {}^{\partial\sigma}/_{\partial U}\right)$ and grand free energies; the only notable difference is that the surface charge at a given potential is different. While the electrode potential inferred from the Fermi level, and hence surface charge, at which electron polarons emerge are not consistent with experiments, the polaron formation potentials from CIP-DFT calculations agree extremely well with experiments, see Fig. 1 and 2. We therefore conclude that the CIP-DFT is more appropriate for treating potential-dependent polaron formation than GCE-DFT.

Although the polaron formation potentials obtained with the CIP-DFT approach align well with the experiments, Supplementary Fig. 1 clearly indicates that within the bandgap, the thermodynamics may not be accurate. This is evidenced by the fact that the free energy as function of the electrode potential is not convex. Furthermore, previous studies have shown that constant potential simulation of reaction kinetics and thermodynamics are not reliable if the Fermi level crosses the bandgap.[18] In our previous work, such behavior was attributed to the finite-size effects of the TiO_2 slab as the semiconductor bulk cannot be reached using just a few atomic layers due to the low charge carrier concentration in semiconductors. For this reason, treating semiconductor surfaces with DFT is very difficult and prone to finite-size effects, and several[19-21]DFT models approximating the semi-infinite bulk as a dielectric continuum and

different neutralizing background treatments on the semiconductor side (space-charge region) have been developed. In all these semiconductor models, the electrode potential can be retrieved from the electrostatic potential in the dielectric continuum. The used CIP-DFT has a few similarities with and differences to these continuum+DFT models:

1) In CIP-DFT the system is neutralized by the electrolyte, not the space-charge region. This basically means that we assume that we have a low concentration electrolyte and highly doped semiconductor such that most of the potential-drop takes place on the electrolyte side. We have confirmed that this is always the case for metallic electrodes but TiO_2 has a small drop (~10-15%) also on the "bulk" semiconductor side. For thick enough slabs also the semiconductor will have the potential drop only on the electrolyte side if the semiconductor is highly doped. Hence, we are neglecting this backside drop and assuming that this is negligible; this introduces an acceptable ~10-15% error in the applied electrode potential. A similar error in the applied potential is often observed in the used dielectric continuum models due to the incorrect electrolyte capacitance.[22]

2) We assume that the dielectric constant of TiO_2 is the same as vacuum. Hence, we use the dielectric permittivity of 1 instead of the experimentally determined dielectric constant 22.[23] This will impact the electrostatic potential decay within the semiconductor "bulk" but is not expected to impact polaron formation potentials or HER thermodynamics and kinetics.

Overall, the CIP-DFT is expected to give a reliable prediction of the polaron formation potentials – this is supported by the good match with experiments. Also, the computed reaction energetics are expected to be at least qualitatively correct and accurate enough to distinguish between the different materials and pathways. However, due to the finite-size effects from the used slab model, CIP-DFT should not be currently used to study reaction pathways where the Fermi level crosses the band gap; in this work we hence present results only for pathways where the Fermi level does not cross the band gap.

S1.2. Vacancy formation calculations

The formation energy of single oxygen vacancy (E_{V_O}) is calculated using the following equation:

$$E_{V_O} = E_{tot} - E_{tot\_V_O} - \frac{n}{2}E_{O_2} \quad (5)$$

where E_{tot} is the total energy of pristine TiO_2 at PZC condition, $E_{tot\_V_O}$ is the total energy of pristine TiO_2 with single oxygen vacancy at PZC and different electrode potentials, E_{O_2} is the energy of oxygen gas molecule and n is the number of oxygen vacancies. The free energy of O_2 was computed as $G(O_2) = 2G(H_2O) - 2G(H_2) - 4.92$ eV from the free energy change of the reaction $O_2 + 2H_2 \rightarrow 2H_2O$, which is 4.92 eV under the standard condition.[11]

S1.3. Calculation of the Tafel slope

The Tafel slope is calculated based on b = (2.3RT)/(αF), where α is obtained from the relation $\Delta G^{\#} = \Delta G_0 - \alpha eU$ and can be extracted as the slope of the potential-dependent reaction energy vs reaction barrier plot, see Supplementary Fig. 25.

Section S2. Additional computational and experimental results

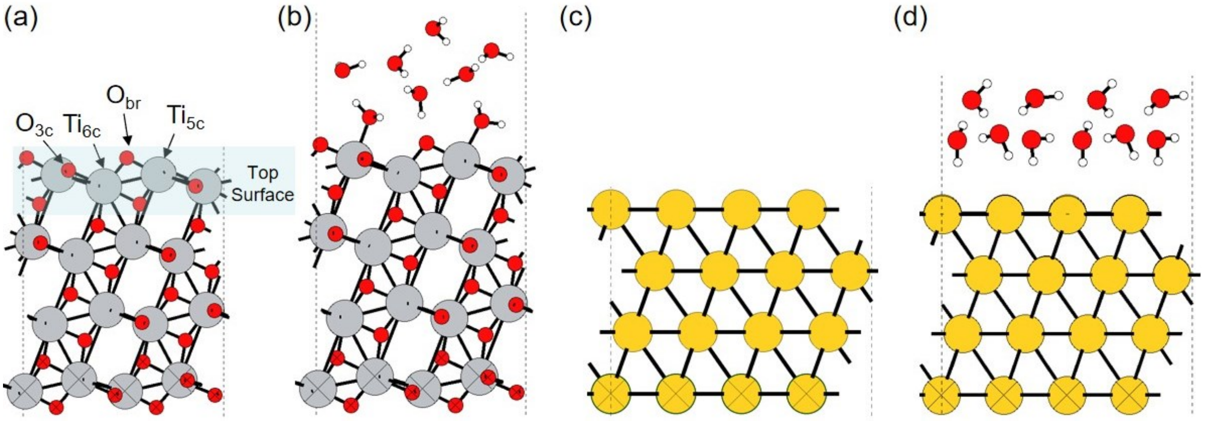

Supplementary Fig. 2 | (a,b) The atom configurations for the TiO_2(101) surface and the water-TiO_2(101) surface. (c,d) The atom configurations for Au(111) and water-Au(111) surfaces. Ti: gray; O: red; H: white; Au: yellow.

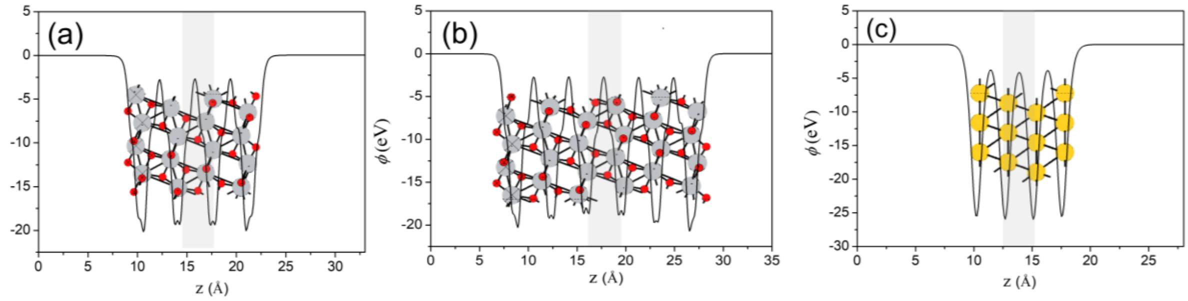

Supplementary Fig. 3 | The xy-averaged electrostatic potentials for (a) TiO_2(101) with four layers, (b) TiO_2(101) with six layers and (c) Au (111), respectively, in which the gray area is the selected inner potential region.

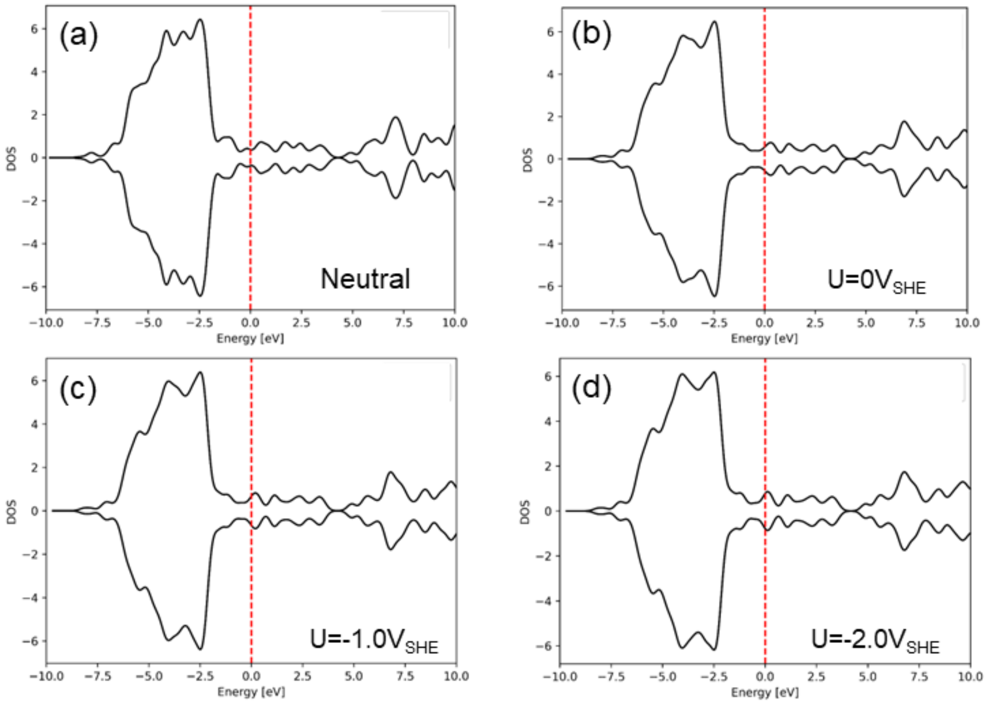

Supplementary Fig. 4 | (a) The electronic density of states (DOS) at constant charge for the Au(111) surface. (b-d) The electronic DOSs as a function of electrode potential for the Au(111) surface.

For Au (111) surface, the DOS shows that the electron states relative to E_f at 0 V_{SHE} have only minor changes compared to that at constant charge (neutral), as shown in Supplementary Fig. 4. Even if larger electrode potentials of -1.0 V_{SHE} and -2.0 V_{SHE} are applied the DOSs remain almost unchanged. This is consistent with the observations for the Pt(111) surface in Liu's work.[15]

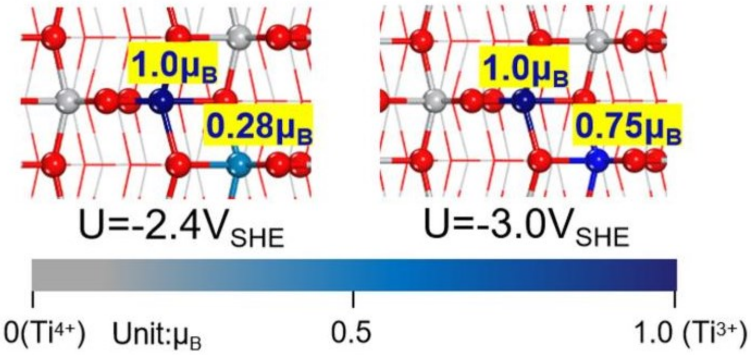

Supplementary Fig. 5 | The AMMs for two reduced Ti atoms on the TiO_2 (101) surface at -2.4 V_{SHE} and -3.0 V_{SHE}, respectively.

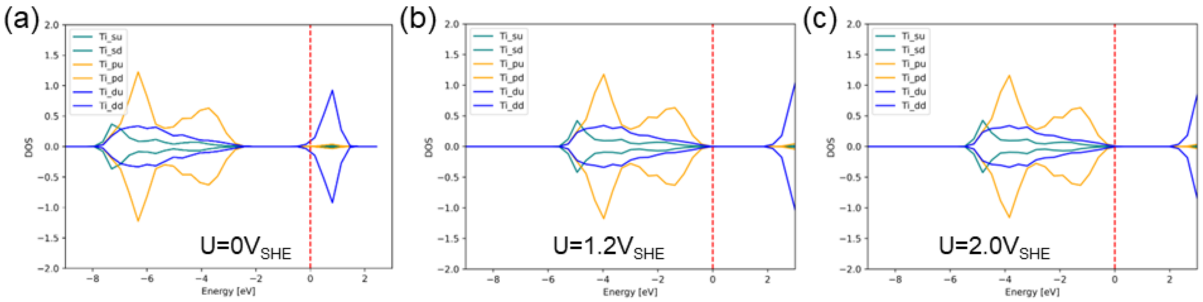

Supplementary Fig. 6 | The electronic density of states (DOS) as function of the electrode potential on TiO_2 (101).

The comparison of PDOS plots in Supplementary Fig. 6 shows that the electronic states at valence-band maximum approach E_f at U = 1.2 V_{SHE}. Increasing the potential further up to U = 2.0 V_{SHE} does not change the number of occupied electronic states relative to E_f. This observation can be attributed to the large number of unoccupied surface states in the conduction band of TiO_2 (101) which leads to strong pinning of the barrier of positive charge at higher positive electrode potentials.

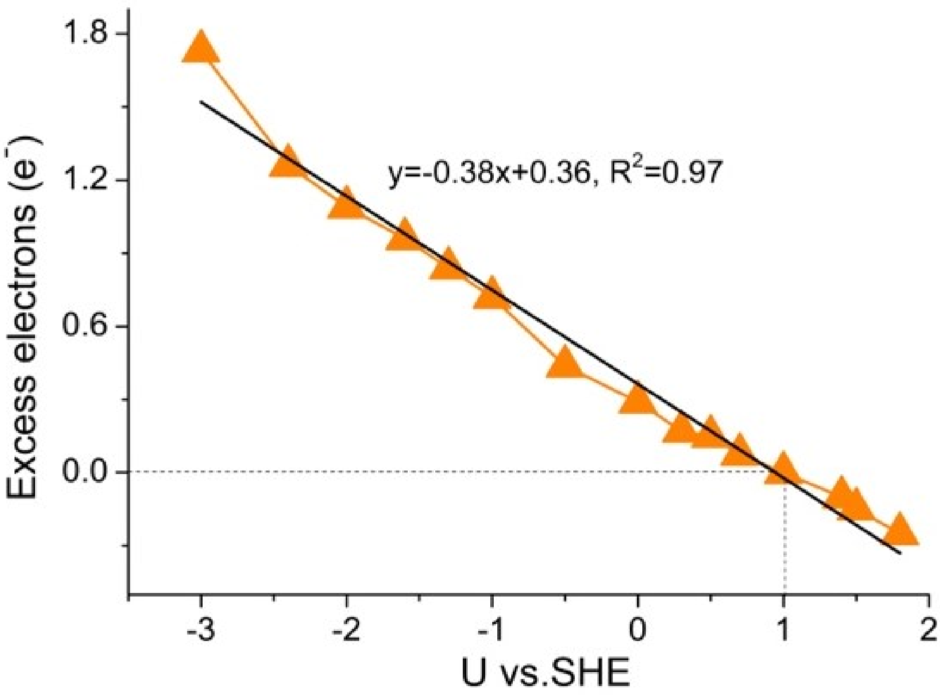

Supplementary Fig. 7 | Change in the number of excess electrons as function of electrode potential.

The potential of zero charge (PZC) was identified by studying how the number of excess electrons (surface charge) in the TiO_2 system varies with the electrode potential. The results in Supplementary Fig. 7 shows that the PZC is located at U = 1.0 V_{SHE}, where the number of excess electrons is zero.

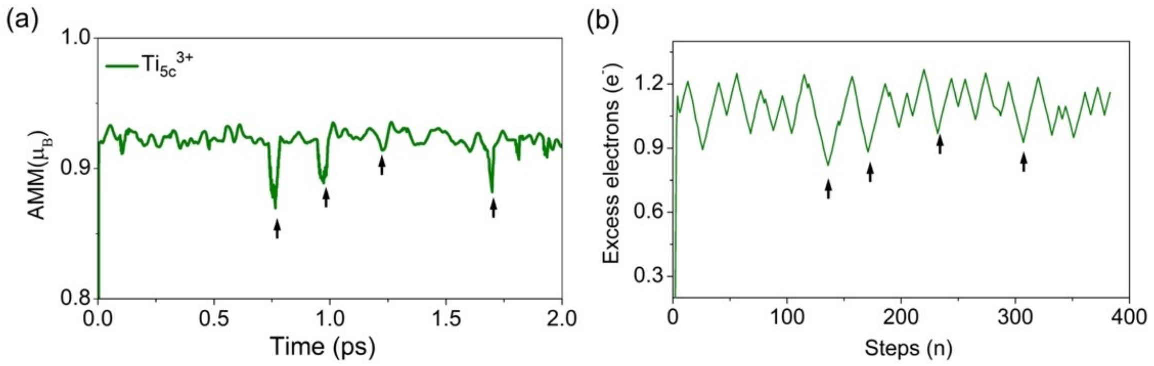

Supplementary Fig. 8 | (a) The variation of AMM for a Ti_{5c}^{3+} polaron state on TiO_2 (101) at U = -2.0 V_{SHE}. The arrows indicate where the AMM value significantly decreases when time varies. (b) The comparison of excess electron variation as a function of time at U = -2.0 V_{SHE}.

By monitoring the AMM of Ti_{5c}^{3+} as a function of time, we observe that Ti_{5c}^{3+} remains stable but exhibits several sudden fluctuations at U = -2.0 V_{SHE}, as indicated by the black arrows in Supplementary Fig. 8a. We would like to note the spikes are rather small in intensity, ~0.1 AMM, and likely result from small fluctuations in the atomic positions and system charge during the constant temperature – constant potential molecular dynamics. We also analyzed the time-dependent surface charge during the CIP-DFT-MD simulation at U = -2.0 V_{SHE} (Supplementary Fig. 8a) and found that that the excess electrons display small fluctuations around the average number of excess electrons (Supplementary Fig. 8b). These fluctuations are normal and expected for any system studied using constant potential MD as the charge fluctuations result from the action of the (computational) potentiostat fixing the electrode potential. See for instance the original CIP-DFT paper.[12]

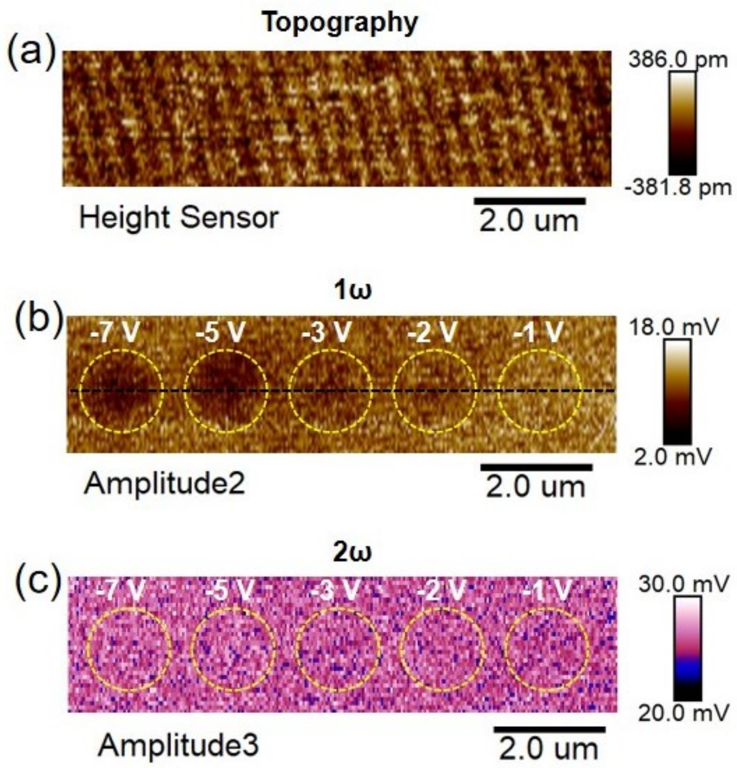

Supplementary Fig. 9 | (a-c) Topography maps (a), EFM-1ω (b) and EFM-2ω (c) measurements on the pristine TiO_2 sample subjected to tip biases in a range of -1 ~ -7V at different locations. Scale bar, 2 μm. The regions of interest are marked by circles.

To verify that the electron aggregation induced by applying a negative electrode potential leads to the formation of Ti^{3+} surface polarons, we conducted *in situ* electrostatic force microscopy (EFM) measurements under atmospheric conditions. This measurement method is highly sensitive to surface charges and can detect the formation of polarons induced by the potential. The EFM topography image in Supplementary Fig. 9a displays a uniform profile of the sample surface after being subjected to a range of tip biases from -1 V to -7 V and suggests that sample has not undergone structural deformations. The EFM 1ω signal mapping in Supplementary Fig. 9b shows that when the TiO_2 sample is exposed to negative tip biases, a noticeable surface charge appears. When the applied voltage is further decreased from -1 V to -2 V, the 1ω signal increases, illustrating the potential-dependent local charge accumulation, see Supplementary Fig. 9b. We ascribe the observed EFM signals to the aggregation of Ti^{3+} polarons on the surface. This assignment is in line with the absence of pronounced features in

the 2ω signal, suggesting that the dielectric constant and thus the stoichiometry of the TiO_2 sample remains unchanged under different electrode potential conditions, see Fig. 9c.

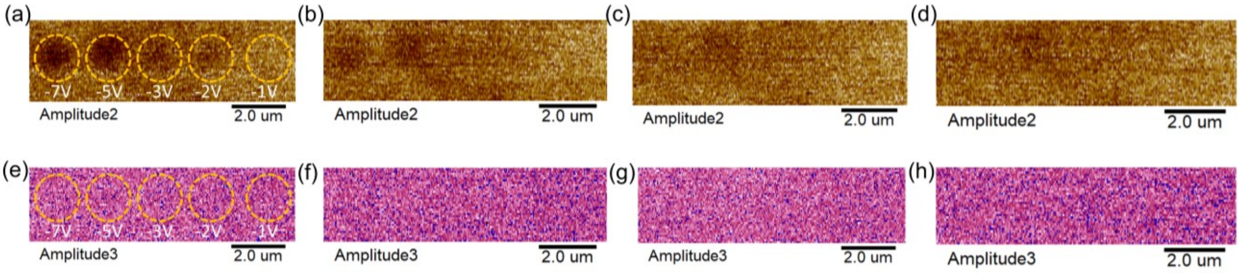

Supplementary Fig. 10 | (a) EFM-1ω observed on the pristine TiO_2 sample subjected to tip biases in a range of -1 ~ -7V at different locations. EFM-1ω observed on the pristine TiO_2 samples under open-circuit conditions at 1 min (b), 3 min (c), and 5 min (d) after voltage removal. (e) EFM-2ω measurements on the pristine TiO_2 sample subjected to tip biases in a range of -1 ~ -7V at different locations. EFM-2ω observed on the pristine TiO_2 samples under open-circuit conditions at 2 min (f), 4 min (g), and 6 min (h) after voltage removal.

The results show that the voltage-driven Ti^{3+} polarons disappear within 1 minutes after the removal of the applied voltage in the range of -1 V to -3 V, as shown in Supplementary Fig. 10b. Moreover, under higher voltages of -5 V and -7 V, the Ti^{3+} polaron disappears within 3 minutes after voltage removal, see Supplementary Fig. 10c and 10d. The absence of pronounced features in the EFM-2ω signal suggests that the dielectric constant and thus the stoichiometry of the TiO_2 sample remains unchanged under different voltage conditions, see Supplementary Fig. 10e-h.

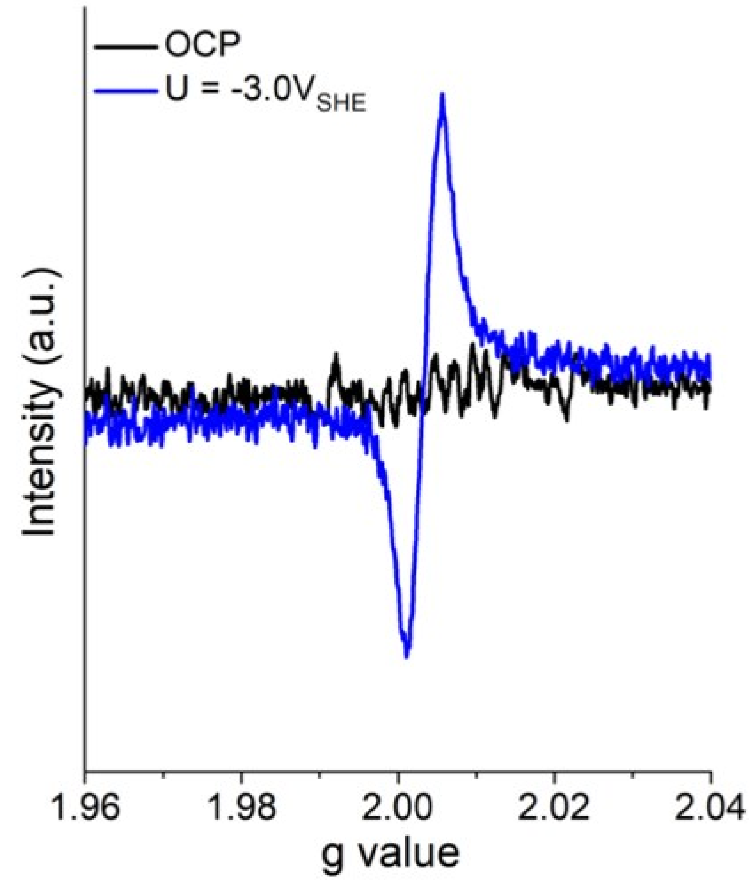

Supplementary Fig. 11 | *In situ* EPR spectroelectrochemical measurements of TiO_2 sample.

In situ EPR spectroscopies demonstrate that the signal of potential-dependent Ti^{3+} polaron formation disappears under open-circuit conditions after removal of the electrode potential at U = -3.0 V_{SHE}, see Supplementary Fig. 11.

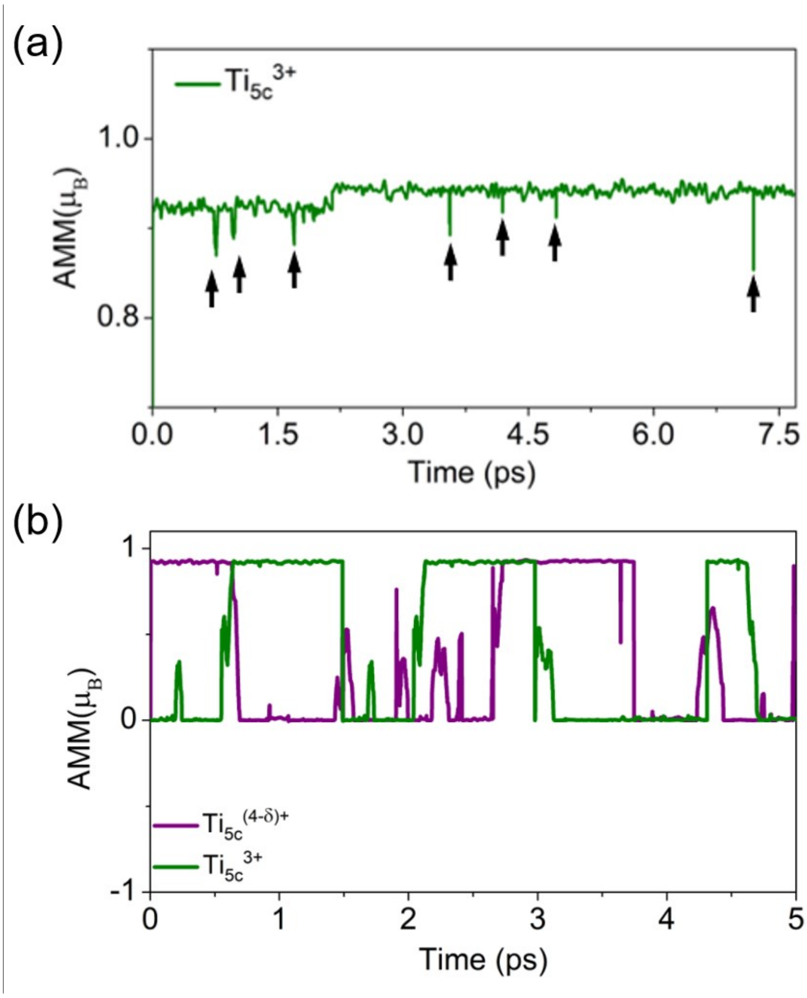

Supplementary Fig. 12 | Extended simulations of (a) the variation of AMM for a Ti_{5c}^{3+} polaron state on TiO_2 (101) at U = -2.0 V_{SHE}. The arrows indicate where the AMM value exhibits the most notable fluctuations. (b) The variation in AMM for Ti_{5c}^{3+}/$Ti_{5c}^{(4-\delta)+}$ polaron states on TiO_2 (101) at U = -2.4 V_{SHE}. δ in the superscript indicates the number of excess electrons ($0 < \delta \leq 1$). The yellow shaded area indicates short time dynamics in Supplementary Fig. 8 .

We extended the simulation time scale and observed that a single potential-dependent Ti^{3+} polaron remains stable over an extended period, whereas multiple coexisting Ti^{3+} polarons exhibit instability, see Supplementary Fig. 12.

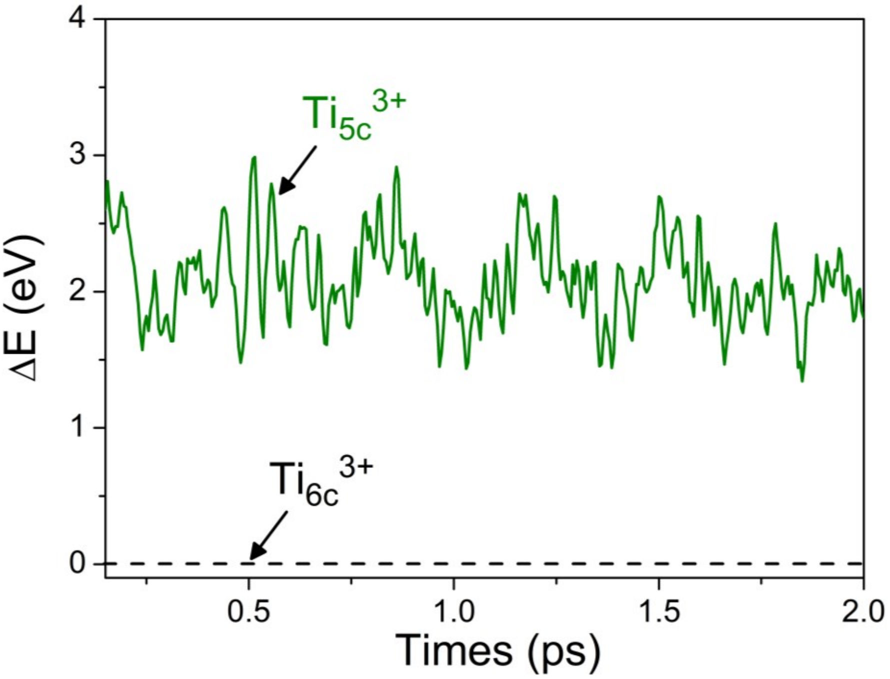

Supplementary Fig. 13 | (a) The dynamic behavior of Ti^{3+} polaron state on V_O-TiO_2 (101) calculated with the CIP-DFT-MD at U = -1.0 V_{SHE} and T = 300 K. The dashed line represents the energy of Ti_{6c}^{3+} cation calculated with the static CIP-DFT approach at U = -1.0 V_{SHE}.

Section S3 Impedance results

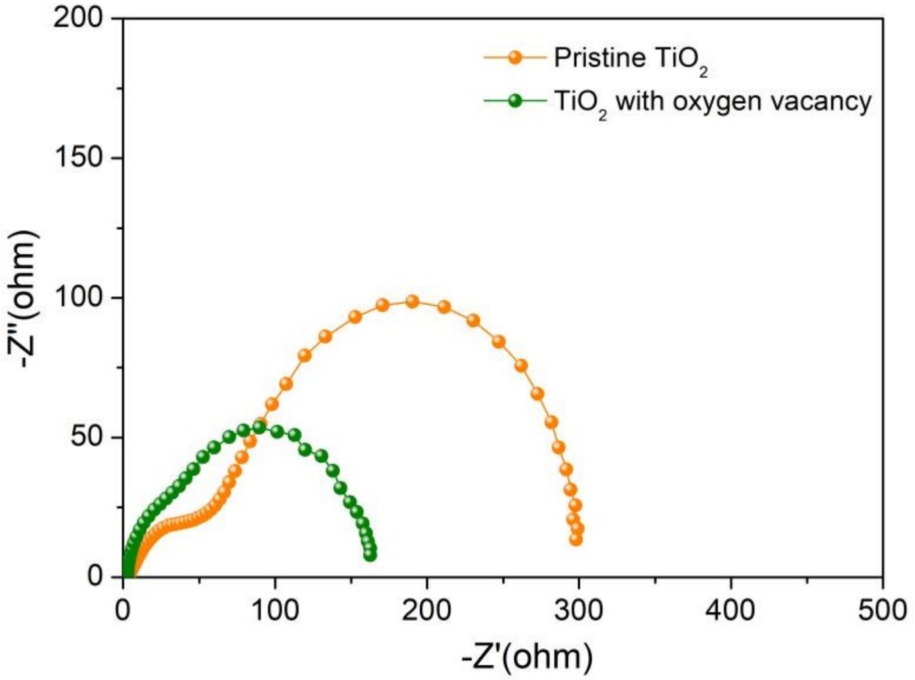

Supplementary Fig. 14 | Electrochemical impedance plots of pristine TiO_2 and oxygen vacancy modified-TiO_2.

Section S4 DOS analysis and magnetic moments

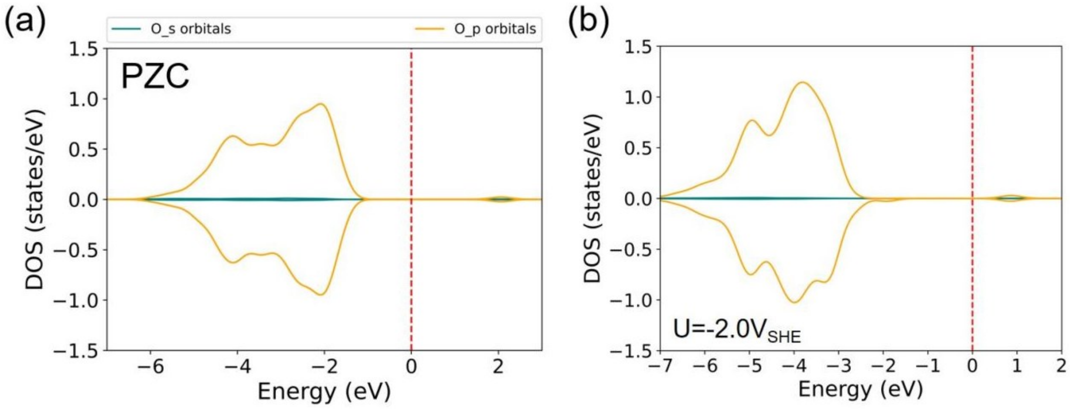

Supplementary Fig. 15 | The PDOS analyses of an O_{br} atom on the TiO_2 (101) surface before and after potential-dependent polaron formation. The plots show that the Fermi level does not cross the electronic levels of an O atom when applying a negative electrode potential.

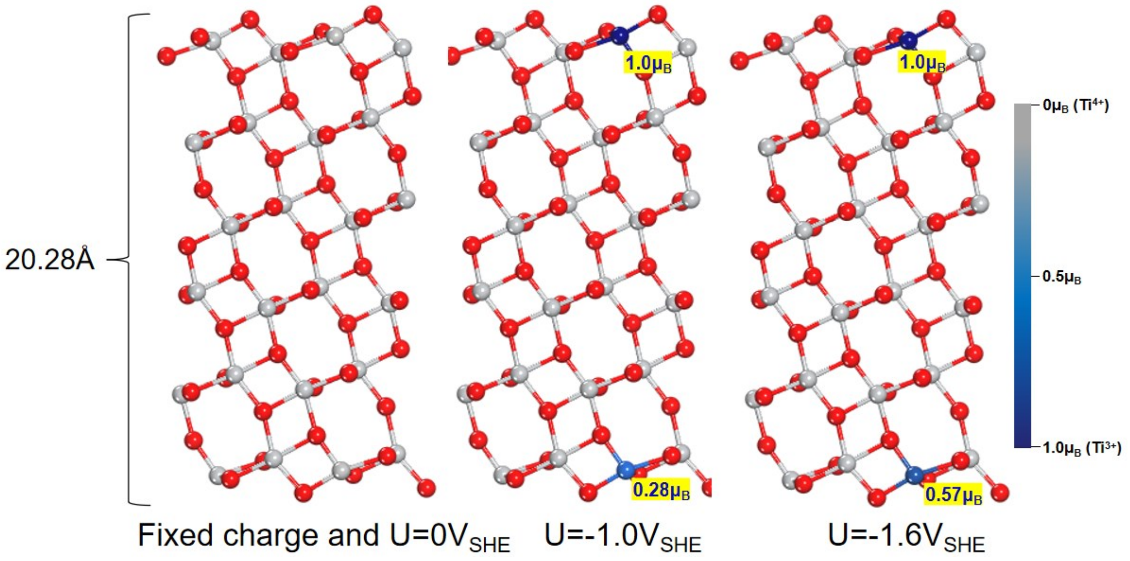

Supplementary Fig. 16 | Potential-dependent AMMs for TiO_2 (101) with six trilayers.

Supplementary Table 2. The comparison between TiO_2 (101) and s-TiO_2 (101) for both charge (q) and AMM (μ_B) as a function of the electrode potential.

	TiO_2 (101)		s-TiO_2 (101)	
Electrode potentials (V_{SHE})	Charge(q)	AMM (μ_B)	Charge(q)	AMM (μ_B)
0	0.29	0	0.39	0
-1.0	0.72	0.74	1.27	1.28
-1.6	0.96	0.91	1.49	1.57

We further examined the effect of thickness of the TiO_2 (101) surface on Ti^{3+} formation and distribution. First, a thicker TiO_2 (101) slab with six-layers (s-TiO_2 (101)) was constructed, corresponding to increasing the thickness from 13.07 Å (TiO_2 (101)) to 20.28 Å (s-TiO_2 (101)), shown in Supplementary Fig. 16. Calculations for the thicker slabs show that at U = 0 V_{SHE} a charge of 0.39 e^- (compared to 0.29 e^- on the thin slab) accumulates on the surface but the AMM remains zero, see Supplementary Fig. 16 and Supplementary Table 2. The corresponding PDOS of a Ti atom also shows that the unoccupied Ti-3*d* states are slightly shifted below the E_f and filled with electrons but the AMM shows no signatures of spin polarization, similar to TiO_2

(101) system at U = 0 V_{SHE}. When the electrode potential is decreased to -1.0 V_{SHE}, 1.27 electrons accumulate on the surface and is significantly higher than 0.72 e^- observed for TiO_2 (101). These excess electrons reduce two surface Ti^{4+} atoms, one to Ti^{3+} polaron state with 1.0 μ_B and another partially reduced Ti^{4+} with 0.28 μ_B (see Supplementary Fig. 16). As the electrode potential is decreased to -1.6 V_{SHE}, the number of excess electrons is further increased to 1.49 e^-, which facilitates the reduction of a surface Ti^{4+} atom to carry an AMM of 0.57 μ_B. These observations confirm that the generated Ti^{3+} polarons primarily localize on the surface of TiO_2, in accordance with the experimental results, which show that the surface conductance of a semiconductor is increased when a negative electrode potential is applied.[24,25]

Section S5 HER mechanism, thermodynamics and kinetics on TiO_2

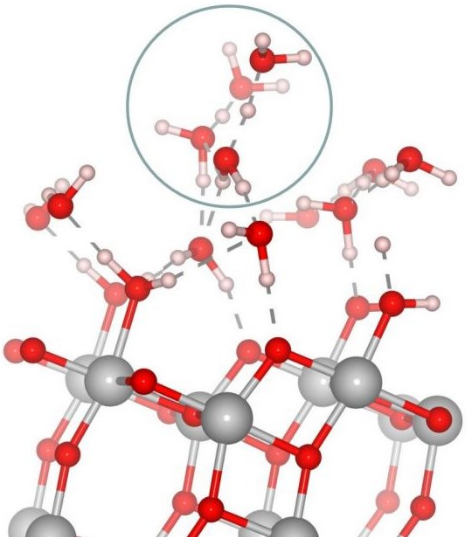

Supplementary Fig. 17 | The atom configuration of proton distribution at U = +2.0 V_{SHE} on the pristine TiO_2 (101).

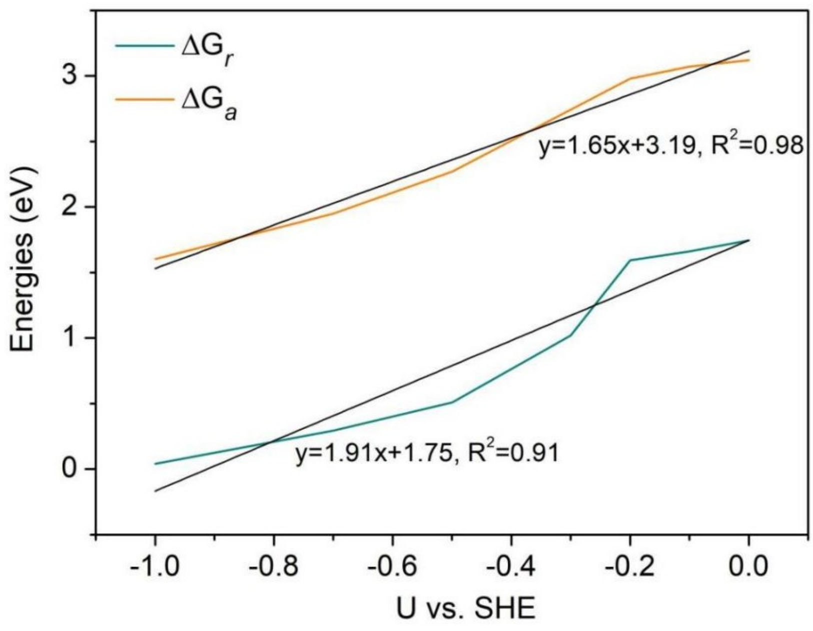

Supplementary Fig. 18 | ΔG_r and ΔG_a on pristine TiO_2 (101) for the Heyrovsky step as a function of electrode potentials.

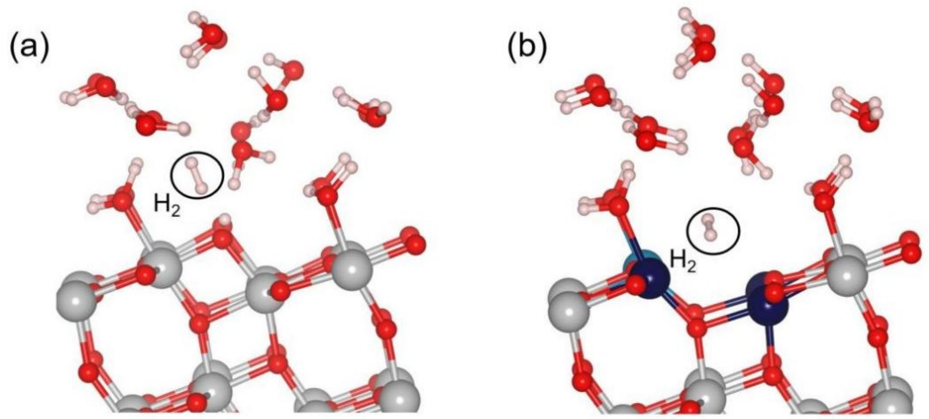

Supplementary Fig. 19 | H_2 formation during the Heyrovsky step on pristine TiO_2 (101) and V_{2O}-TiO_2 (101) surfaces.

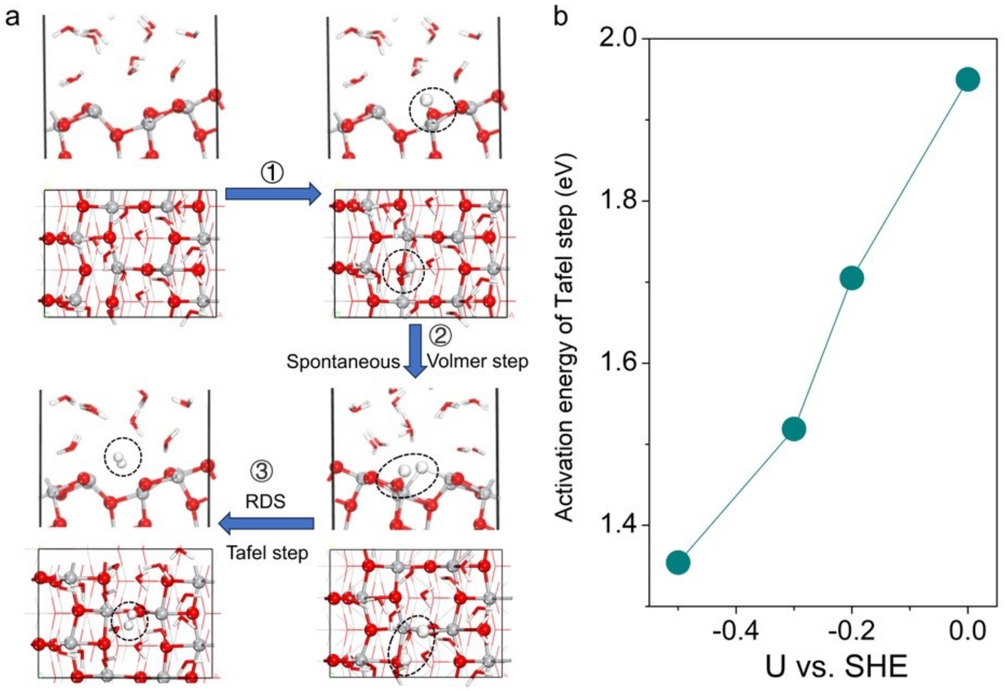

Supplementary Fig. 20 | (a) A HER process on the V_{2O}-TiO_2 surface. (b)The activation energy of a Tafel step as a function of a potential on the V_{2O}-TiO_2 surface.

On the V_{2O}-TiO_2 surface, surface oxygen atoms readily interact with H atoms to form hydroxyl group and we examined the adsorption of a hydrogen atom on the oxygen atom nearest to the oxygen vacancy, which leads to OH group without a barrier through a Volmer step ① in Supplementary Fig. 20a. Our CIP-DFT structure relaxation and NEB results indicate that the second Volmer step involves the nearest oxygen atom adjacent to the vacancy, forming an OH functional group. This process leads to the spontaneous, barrierless dissociation of H_3O^+ to yield a geometry where a hydrogen atom directly adsorbs at the V_O site located between two neighboring Ti^{3+} atoms. This step is depicted as step ② in Supplementary Fig. 20a.

Subsequently, the two adsorbed hydrogen atoms combine to form molecular hydrogen via the "chemical" Tafel step (see step 3 in Supplementary Fig. 20a), which exhibits a relatively high and strongly potential-dependent barrier (see Supplementary Fig. 20b); we identify the Tafel step as the rate-determining step (RDS) on V_{2O}-TiO_2. Overall, our simulations support the Volmer-Tafel mechanism as the operative HER pathway, where $*H + * + H^+ + e^- \rightarrow *H + *H$ step is fast and pre-equilibrated, while $*H + *H \rightarrow * + H_2$ is the rate-determining "chemical" step.

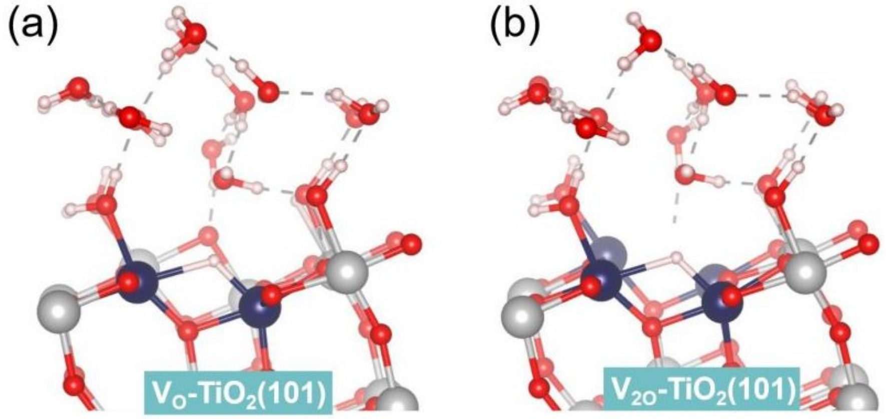

Supplementary Fig. 21 | (a) The atom configuration of H^+ adsorption on a V_O-site in V_O-TiO_2(101). (b) The atom configuration of H^+ adsorption on a V_O-site in V_{2O}-TiO_2 (101).

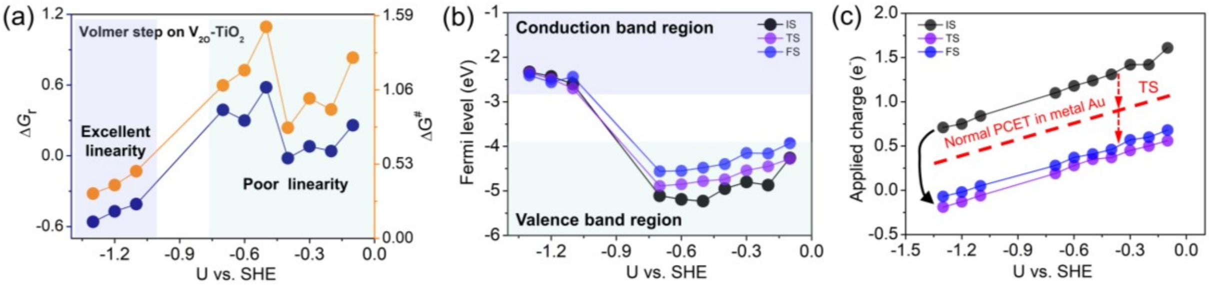

Supplementary Fig. 22 | (a) Grand canonical reaction free energies (dark blue) and activation free energies (yellow) for Volmer step on a V_{2O}-site at the TiO_2 (101) with the double oxygen vacancy. (b) The changes of Fermi level of IS, TS and FS as a function of electrode potential. (c) The surface charges of IS, TS and FS of first Volmer reaction at the respective potentials on V_{2O}-TiO_2 (101). The dark red dash line represents the common proton-coupled electron transfer behavior in metal Au electrode.

Supplementary Fig. 22a shows that the Volmer reaction energies (ΔG_r) and the activation energies (ΔG_a) on the V_{2O}- exhibit piecewise-linearity as a function of electrode potential. This behavior is characterized by a region of poor linearity at slightly reducing potentials and robust linearity at highly reducing potentials, see Supplementary Fig. 22a. At slightly reducing potentials, the number of Ti^{3+} polaron increases as electrode potential is made more reducing while at highly reducing potentials the number of polarons remains constant, see Supplementary Fig. 22 and Supplementary Table 7. This can be understood based on the results in Supplementary Fig. 22b, which shows that the Fermi level is always at the valence band at slightly reducing potentials and moves to the conduction band at highly reducing potentials. These potential-dependent electronic structure changes confirm that the number of Ti^{3+} polarons increases as a function of electrode potential when Fermi level localizes at the valence band. In this potential region, a poor linearity of the Volmer barrier is observed. However, when the Fermi level is within the conduction band, the number of polarons remains constant and the Volmer barrier varies linearly. Thus, we conclude that the poor linearity between the potential and reaction energy is due to the potential-dependent Ti^{3+} polaron formation at potentials -0.7 – 0.0 V vs RHE. This observation is qualitatively different from fully linear behavior observed for metallic electrodes in general.[26]

By analyzing the potential-dependent charge transfer along the Volmer reaction pathway on the V_{2O}-TiO_2 surface, we observe that as the reaction proceeds from the transition state to the final state, the surface loses electrons and becomes more positively charged. At the transitions state, the system carries a more negative charge than at the final state (Supplementary Fig. 22c). Thus, the number of electrons in the system varies non-linearly when the system proceeds from the initial (IS) to transition (TS) and to final states (FS) at a constant electrode potential in TiO_2 system. This behavior is qualitatively different from that observed for the Au(111), where the transition state is more positively charged than the final state (Supplementary Fig. 22c red dash line), exhibiting monotonous linear increase as the reaction proceeds from IS to FS. The non-linear charge transfer further suggests that the proton-electron transfer process in TiO_2 system is partially decoupled or asynchronous as electron transfer precedes proton transfer while simultaneous proton-coupled electron transfer (PCET) is observed on various metal electrodes.[14,15] The comparison between semiconducting V_{2O}-TiO_2 and metallic Au highlights that the potential-dependent electronic structure or polaron formation makes charge transfer and reaction mechanisms of a semiconductor electrode qualitatively different from those of a metallic electrode.

Overall, our computational results clearly demonstrate that the potential- and structure-dependent formation and concentration of Ti^{3+} polarons play a crucial role on rationalizing activation energies of both Volmer step and Heyrovsky step and achieving electrocatalytic activity on TiO_2. Additionally, oxygen vacancies lead to the accumulation of positive charge, which hinders proton trapping as stable OH_{ads} species and facilitates the Heyrovsky step. We also find that the HER thermodynamics and kinetics on TiO_2 are substantially more complex than on metallic electrodes. Specifically, the reaction energetics exhibit piecewise-linear change as a function of the electrode potential when the polaron concentration remains constant. However, varying polaron concentrations leads to non-linear, unsystematic dependency between the electrode potential and reaction energies. Consequently, we find that the linear free energy relationships between reaction energies and kinetics as a function of potential do not hold for TiO_2 highlighting the need to explicitly study potential-dependent reaction kinetics in (semiconductor) electrocatalysis.

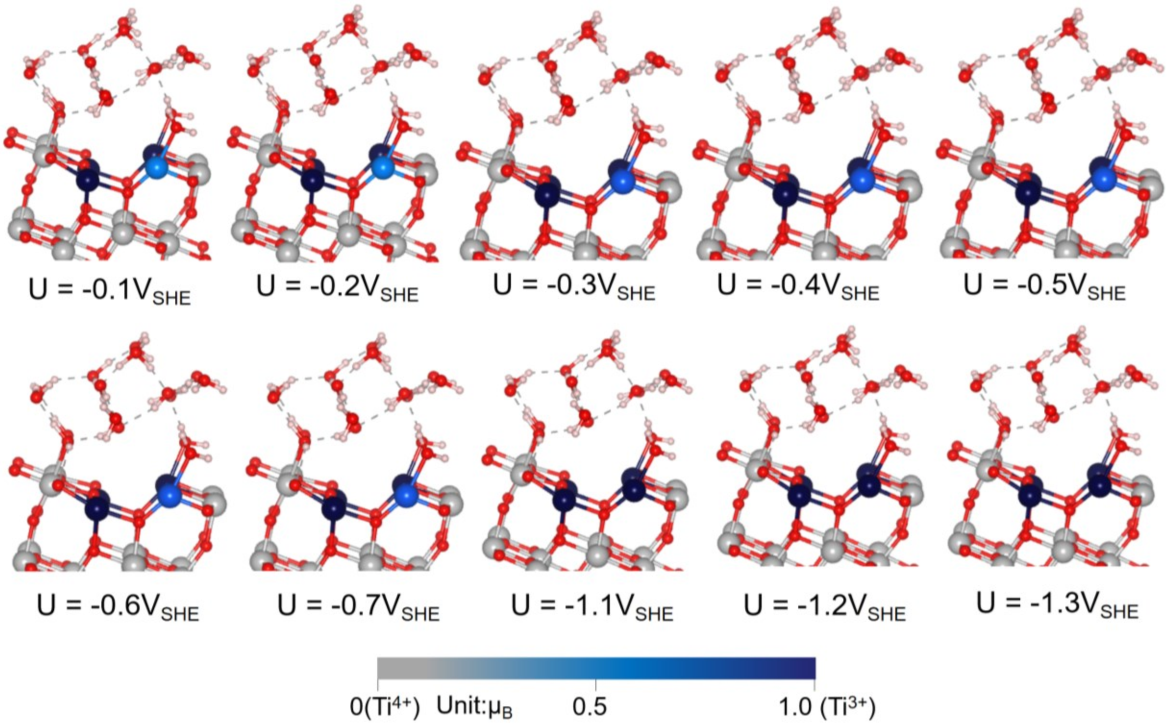

Supplementary Fig. 23 | The AMMs of Ti atoms in the initial state of Volmer reaction on V_{2O}-TiO_2(101) as a function of electrode potential.

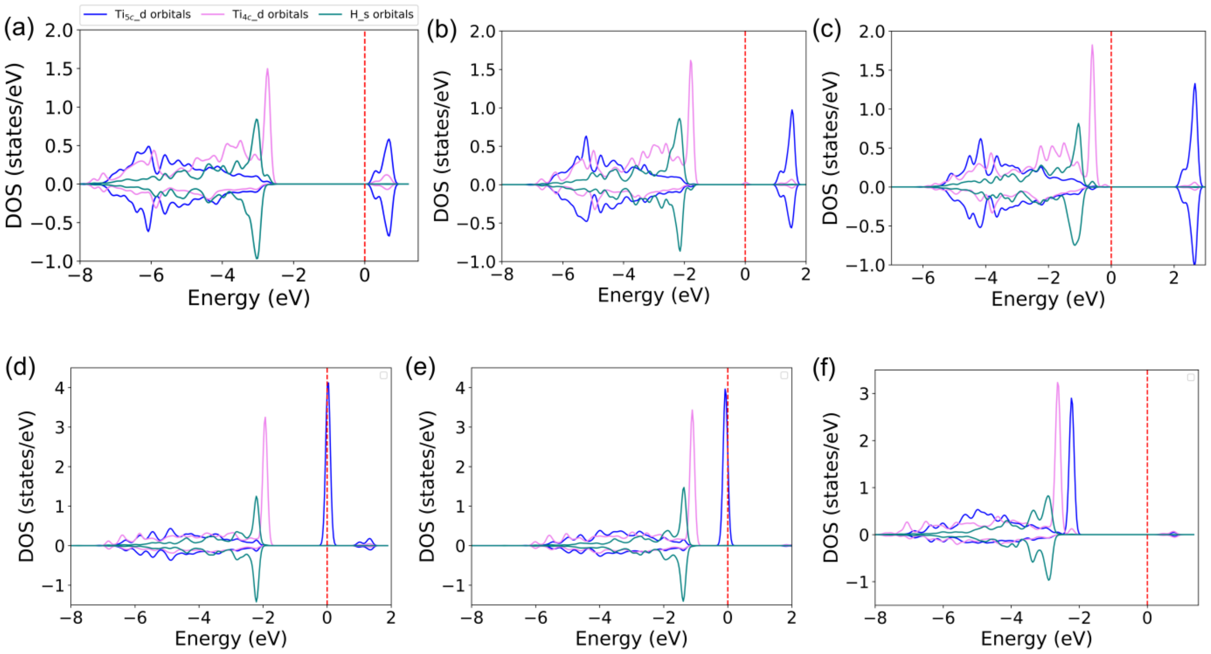

Supplementary Fig. 24 | The PDOS of Ti_{4c}_d, Ti_{5c}_d and H_s after H adsorption on V_{2O}-site in V_{2O}-TiO_2 (101) at (a) U=0 V_{SHE}, (b) U= -0.3 V_{SHE} and (c) U= -1.0 V_{SHE}. The PDOS of Ti_{4c}_d, Ti_{5c}_d and H_s after H adsorption on V_O-site in V_O-TiO_2 (101) at (d) U=0 V_{SHE}, (e) U= -0.3 V_{SHE} and (f) U= -1.0 V_{SHE}.

The DOS analyses of V_{2O}-TiO_2 (101) highlight the significant orbital hybridization between H_s and Ti_{4c}_d electrons for a wide range of energy region below the Fermi level at U = 0 V_{SHE}, see Supplementary Fig. 24a. At more reducing potential of U = -0.3 V_{SHE}, the orbital hybridization between H_s and Ti_{4c}_d shifts toward the Fermi level (Supplementary Fig. 24b), whereas at higher reducing potential of U = -1.0 V_{SHE} it is just below to the Fermi level (Supplementary Fig. 24c), suggesting that the Ti-H bonding strengthens as a function of electrode potential. However, on V_O-TiO_2 (101) the electronic states of Ti_{4c}_d are more localized than those on V_{2O}-TiO_2 (101) at U = 0 V_{SHE} and have no obvious orbital hybridization between H_s and Ti_{4c}_d, see Supplementary Fig. 24d. Moreover, this orbital hybridization does not exhibit any systematic variations at more reductive potentials, see Supplementary Fig. 24e and f. Thus, we conclude that the facilitated Volmer reaction kinetics on V_{2O}-TiO_2 (101) is attributed to the increased orbital hybridization

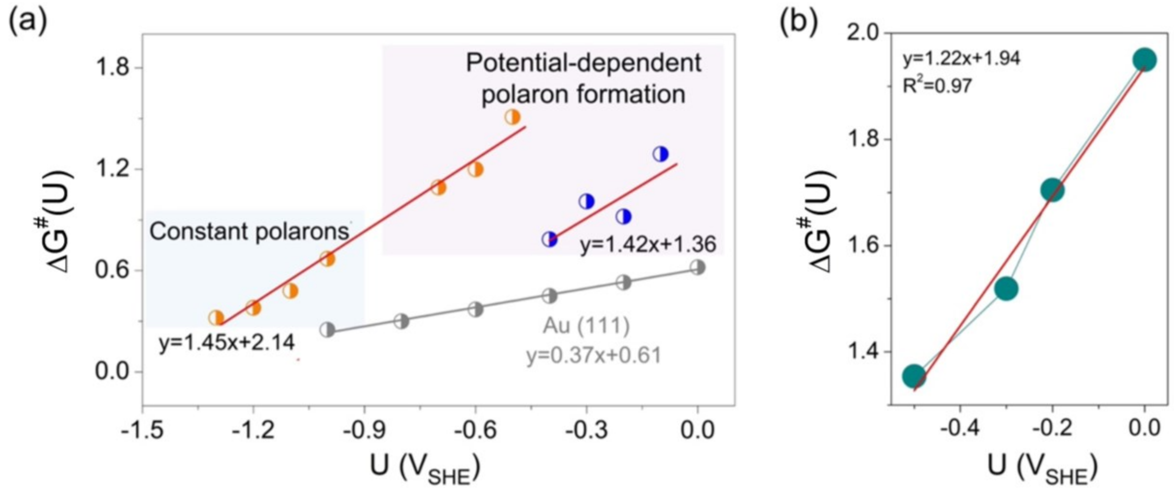

Supplementary Fig. 25 | (a) The grand canonical barriers for Volmer reaction as a function of electrode potential on V_{2O}-TiO_2 and Au. The orange (blue) dots correspond to the second (first) Volmer step. It is important to note that this result stems from the Volmer reaction, which was performed without considering the hydrogen coverage around the V_O site. (b) The grand canonical barriers for the Tafel step as a function of electrode potential on V_{2O}-TiO_2 considering the hydrogen coverage around the V_O site.

We calculated the transfer coefficients of the Volmer step and the Tafel step. According to the formula $\Delta G^{\#} = \Delta G_0 - \alpha eU$, where α is the transfer coefficient, we found that for the Volmer step, α = 1.42 and 1.45 for the first and second Volmer step. (Supplementary Fig. 25a) . For the Tafel step, α =1.22, see Supplementary Fig. 25b. These results suggest that the electrode potential plays a crucial role in lowering the barrier for both the electrochemical Volmer step and chemical Tafel step. Meanwhile, based on b = (2.3RT)/(αF), we obtained a Tafel slope of 48.5 mV/dec, which is close to the experimental value. However, α =1.22 is beyond the scope of traditional theory (0<α<1) and requires further verification of its physical significance.

The underlying reason for the transfer coefficient α to be larger than one is the concomitant and potential-dependent polaron formation and electron transfer in HER steps. This suggests that future research on the mechanism of semiconductor electrocatalysis would benefit from incorporating more systematic analysis.

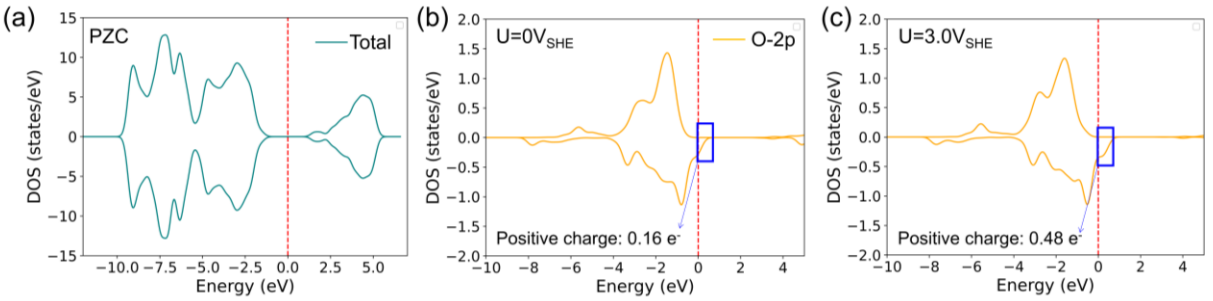

Supplementary Fig. 26 | (a) The total DOS of the NiO (100) surface at PZC of -1.0 V_{SHE}. The PDOS of surface O atom on NiO (100) respective at (b) U = 0 V_{SHE} and (c) U = 3.0 V_{SHE}.

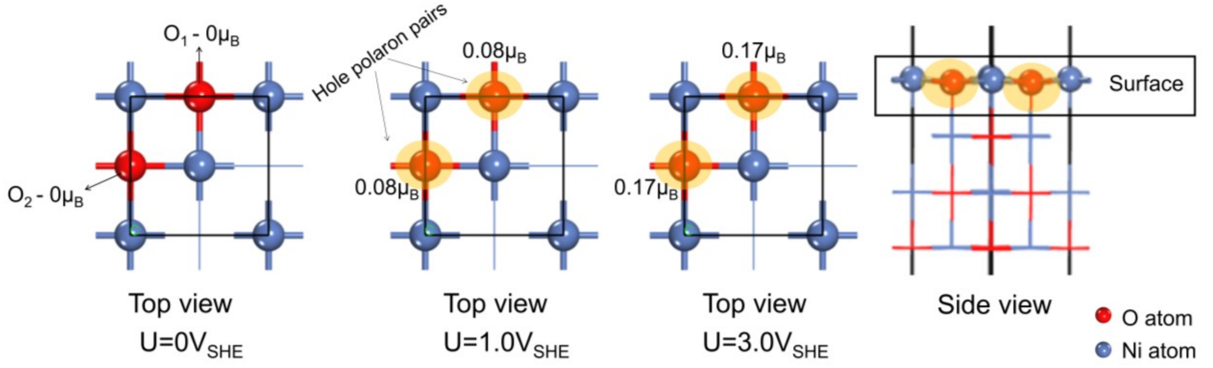

Supplementary Fig. 27 | The hole polaron formation on NiO (100) as a function of electrode potential.

We also observe the formation of potential-dependent hole polaron states on a prototypical p-type NiO semiconductor. It is observed that the Fermi level localizes at the middle of the band gap on NiO semiconductor at PZC of -1.0 V_{SHE}, see Supplementary Fig. 26a. At U = 0 V_{SHE}, the NiO (100) surface carries a positive charge of 0.16 e^-, and the PDOS plot displays that initially occupied O-2p states shift up in energy being partially above E_f, see Supplementary Fig. 26b. However, no AMM are observed on surface O of NiO (100), see Supplementary Fig. 278. Further increasing electrode potential up to U = +3.0 V_{SHE} increases the accumulation of positive charge to 0.48 e^-, shifting the occupied O-2p orbitals more pronouncedly above the Fermi level, see Supplementary Fig. 26c. The increased positive charge from 0 V_{SHE} to 3.0 V_{SHE} is 0.31 e^- and a considerable AMM of 0.34 μ_B residing on double O atoms on NiO (100), makes the more initially occupied O-2p orbitals above the Fermi level, see Supplementary Fig. 26 and 27. AMM in the single O anions is now ~0.17 μ_B and suggests that O^{2-} is partially oxidized with

a clear hole polaron state. Note that the hole polarons prefer to appear as pair on surface (see Supplementary Fig. 27), which is very similar with recent work[27] from Piccinin's group which demonstrates that high photoelectrochemical water splitting activity results from material's capability to form multiple hole polarons in haematite. In TiO_2, electron polarons tend to form the first polaron and then the second polaron sequentially. It discloses that electrons and holes have very different mechanisms for forming polarons, including the number and order of the formations. Furthermore, it can be seen that the hole polaron pairs on O atom exhibits drastic oscillation changes for AMM at $U = +3.0\ V_{SHE}$ on NiO (100), see Supplementary Fig. 28. This drastic oscillation is consistent with that of TiO_2 system with the electron polaron pairs. As a result, advanced CIP-DFT methods are essential for observing hole or electron polarons generation in semiconductor electrode, which is a key step in guiding future experiments and theoretical community in mining novel semiconductor electrocatalysts.

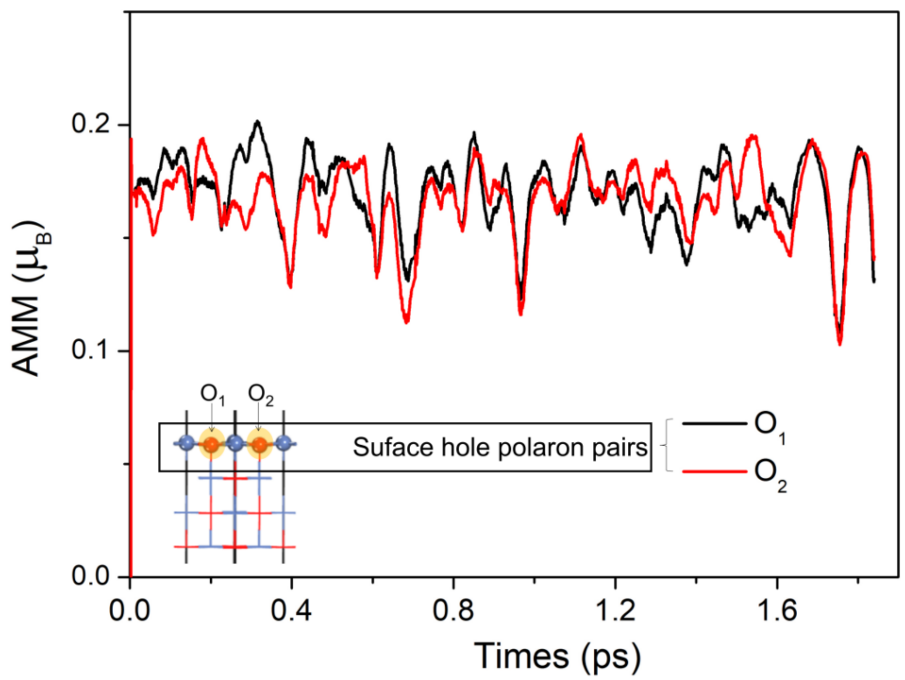

Supplementary Fig. 28 | The dynamic behavior of hole polaron states on NiO (100) surface calculated by using dynamic CIP-DFT-MD approach.

Supplementary Table 3. The reaction and activation energies for the Heyrovsky reaction on pristine TiO_2(101) corresponding to Supplementary Fig. 18, and corresponding surface charges of IS, TS and FS.

Electrode potentials (V_{SHE})	ΔG_r	ΔG_a	Excess electrons (e^-)		
			IS	TS	FS
0	1.747	3.12	1.21	0.48	0.48
-0.1	1.661	3.07	1.15	0.37	0.45
-0.2	1.594	2.98	1.05	0.33	0.35
-0.5	0.509	2.27	1.14	0.22	0.09
-1.0	0.041	1.60	0.79	-0.25	-0.19

Supplementary Table 4. The reaction and activation energies for the first Volmer reaction on O-site in V_O- TiO_2(101), and corresponding surface charges of IS, TS and FS.

Electrode potentials (V_{SHE})	ΔG_r	ΔG_a	Excess electrons (e^-)		
			IS	TS	FS
0	-0.89	0.30	1.18	1.23	1.16

Supplementary Table 5. The reaction and activation energies for second Volmer reaction on Vo-site in V_O-TiO_2(101), and corresponding surface charges of IS, TS and FS.

Electrode potentials (V_{SHE})	ΔG_r	ΔG_a	Excess electrons (e^-)		
			IS	TS	FS
0	0.50	2.03	1.41	0.97	0.56

Supplementary Table 6. The reaction and activation energies for the Volmer reaction on V_{2O}-TiO_2(101), and corresponding surface charges of IS, TS and FS. This part of the results originate from the Volmer reaction, which was conducted without considering the hydrogen coverage surrounding V_O site.

Electrode potentials (V_{SHE})	ΔG_r	ΔG_a	Excess electrons (e^-)		
			IS	TS	FS
-0.1	0.25	1.29	1.61	0.56	0.68
-0.2	0.03	0.92	1.42	0.50	0.60
-0.3	0.07	1.01	1.42	0.45	0.57
-0.4	-0.02	0.78	1.31	0.37	0.46
-0.5	0.58	1.51	1.24	0.35	0.41
-0.6	0.30	1.2	1.18	0.28	0.37
-0.7	0.39	1.09	1.10	0.19	0.28
-1.1	-0.41	0.48	0.84	-0.06	0.05
-1.2	-0.47	0.38	0.75	-0.13	-0.02
-1.3	-0.56	0.32	0.71	-0.19	-0.07
neutral	0.33	0.59			

Supplementary Table 7. The reaction and activation energies for Heyrovsky reaction on V_{2O}-TiO_2(101), and corresponding surface charges of IS, TS and FS.

Electrode potentials (V_{SHE})	ΔG_r	ΔG_a	Excess electrons (e^-)		
			IS	TS	FS
-0.3	0.12	1.10	1.12	1.02	0.57
-1.2	-0.89	0.88	0.54	0.33	-0.01

Supplementary Table 8. Variations in E_f, charge, and AMM with electrode potential for the Volmer step on V_{2O}-TiO_2. It can be seen that the Ti^{3+} polaron exhibits a potential-dependent formation with increasing negative electrode potential from -0.1 V_{SHE} to -0.7 V_{SHE} respective at IS, TS and FS.

Electrode potentials (V_{SHE})	IS			TS			FS		
	E_f	Charge	AMM	E_f	Charge	AMM	E_f	Charge	AMM
-0.1	-4.26	-1.61	1+1+1+0.40	-4.28	-0.56	1+1+0.46	-3.93	-0.68	1+1+0.32
-0.2	-4.87	-1.42	1+1+1+0.61	-4.45	-0.50	1+1+0.53	-4.16	-0.60	1+1+0.42
-0.3	-4.80	-1.42	1+1+1+0.61	-4.54	-0.45	1+1+0.58	-4.15	-0.57	1+1+0.46
-0.4	-4.95	-1.31	1+1+1+0.72	-4.74	-0.37	1+1+0.67	-4.40	-0.46	1+1+0.56
-0.5	-5.23	-1.24	1+1+1+0.8	-4.78	-0.35	1+1+0.69	-4.48	-0.41	1+1+0.62
-0.6	5.19	-1.18	1+1+1+0.86	-4.85	-0.28	1+1+0.75	-4.55	-0.37	1+1+0.67
-0.7	-5.11	-1.10	1+1+1+0.91	-4.89	-0.19	1+1+0.83	-4.56	-0.28	1+1+0.74
-1.1	-2.6	-0.84	1+1+1+1	-2.7	0.06	1+1+1	-2.44	-0.05	1+1+1
-1.2	-2.43	-0.75	1+1+1+1	-2.49	0.13	1+1+1	-2.56	0.02	1+1+1

-1.3	-<br>2.33	-0.71	1+1+1+1	-2.35	0.19	1+1+1	-<br>2.41	0.07	1+1+1

Section S6 Vacancy formation energies

Supplementary Table 9. The formation energies of an oxygen vacancy (E_{V_O}) on TiO_2(101) at PZC and different electrode potentials at the GGA+U level.

Electrode potentials (V_{SHE})	E_{V_O}(unit:eV)	Charge
PZC	2.97	0
0	2.73	-0.26
-0.5	2.56	-0.67
-1.0	1.87	-1.04
-2	0.68	-1.81

Supplementary Table 9 shows that the E_{V_O} is smaller at U = 0 V_{SHE} compared to the value at PZC conditions. As the electrode potential is decreased from 0 V_{SHE} to -2.0 V_{SHE}, the negative surface charge of TiO_2 (101) increases linearly and the oxygen vacancy formation energies decrease linearly. The facilitated oxygen vacancy formation at more reducing potentials can be attributed to the fact that the negatively charged V_O-TiO_2 (101) surface gets stabilized by the positive charge left behind by the oxygen, which brings the slab closer to a charge neutral state.[28] Therefore, we conclude that the potential-driven surface charges play a significant role in facilitating oxygen vacancy formation.

Section S7 SEM, TEM, XRD, XPS and Raman results

The scanning electron microscopy (SEM) images reveal the formation of nanoarray for both pristine TiO_2 and V_O-TiO_2, as shown in Supplementary Fig. 29a and 29d. The transmission electron microscopy (TEM) images of pristine TiO_2 and V_O-TiO_2 are shown in Supplementary Fig. 29b and 29e, respectively. High-resolution TEM (HRTEM) images of both pristine TiO_2 and V_O-TiO_2 exhibit lattice fringes with interplanar distances of 0.351 nm and 0.353 (Supplementary Fig. 29b and 29e inset), respectively, and are attributed to the (101) plane of anatase TiO_2. Many dislocations and distortions can be observed in Supplementary Fig. 29e (open circle), which implies the formation of a defect-rich structure. Energy dispersive X-ray (EDX) elemental mapping images show a uniform distribution of Ti and O elements in both pristine TiO_2 and V_O-TiO_2 nanoarrays, see Supplementary Fig. 29c and 29f.

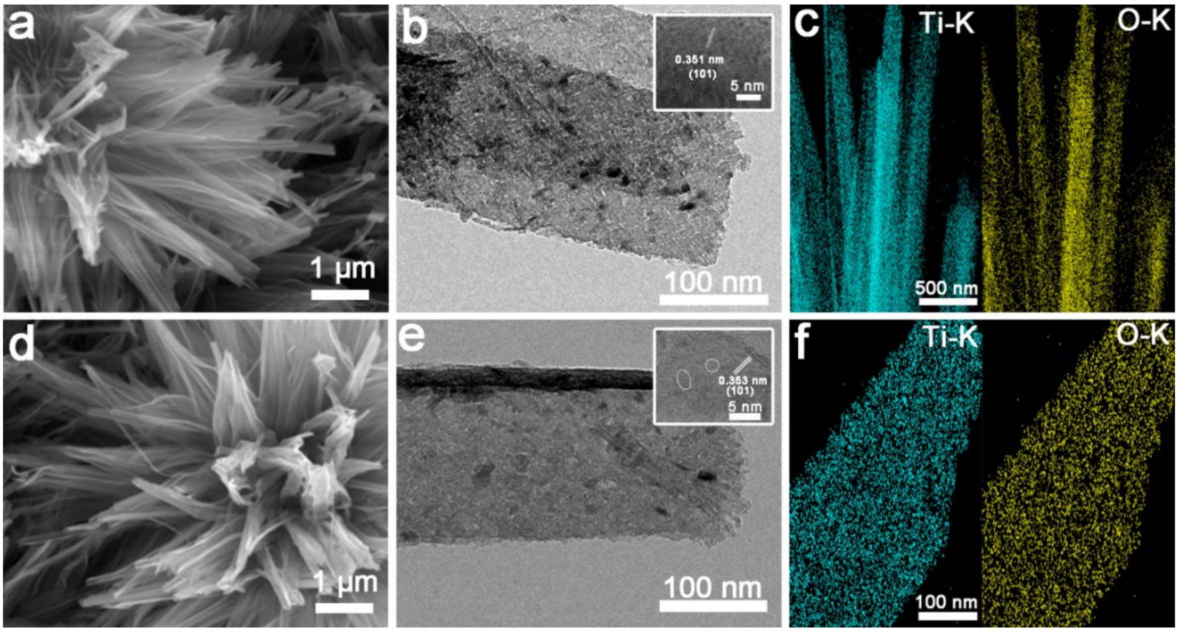

Supplementary Fig. 29 | (a,d) SEM images of pristine TiO_2 and V_O-TiO_2, (b,e) TEM images of pristine TiO_2 and V_O-TiO_2, and (c,f) corresponding EDX elemental mapping images of pristine TiO_2 and V_O-TiO_2.

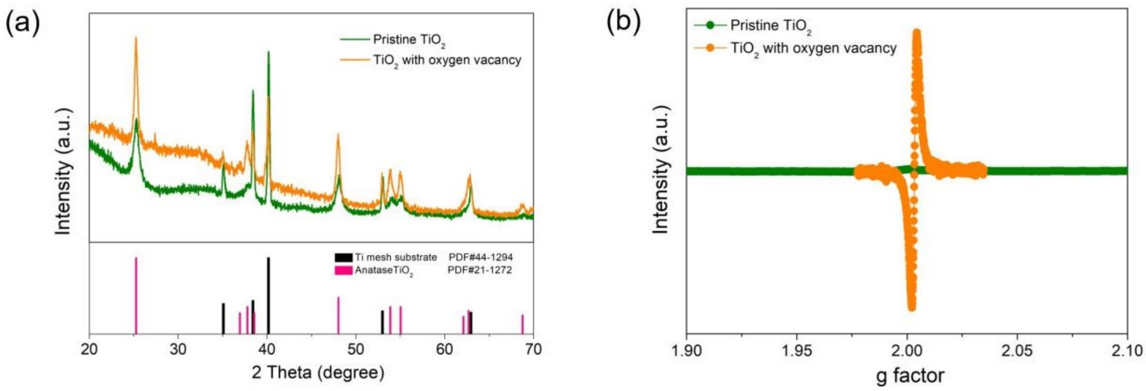

Supplementary Fig. 30 | (a) XRD patterns of both pristine TiO_2 and oxygen vacancy modified-TiO_2. (b) EPR spectra of both pristine TiO_2 and oxygen vacancy modified-TiO_2 measured at 100 K.

The X-ray diffraction (XRD) patterns of pristine TiO_2 and V_O-TiO_2 in Supplementary Fig. 30a show that anatase phase of TiO_2 has been synthesized. Electron paramagnetic resonance (EPR) measured at 100 K shows (Supplementary Fig. 30b) a signal at g = 2.004 in oxygen vacancy modified-TiO_2 catalyst, which confirms V_O defects induce Ti^{3+} polaron.[29]

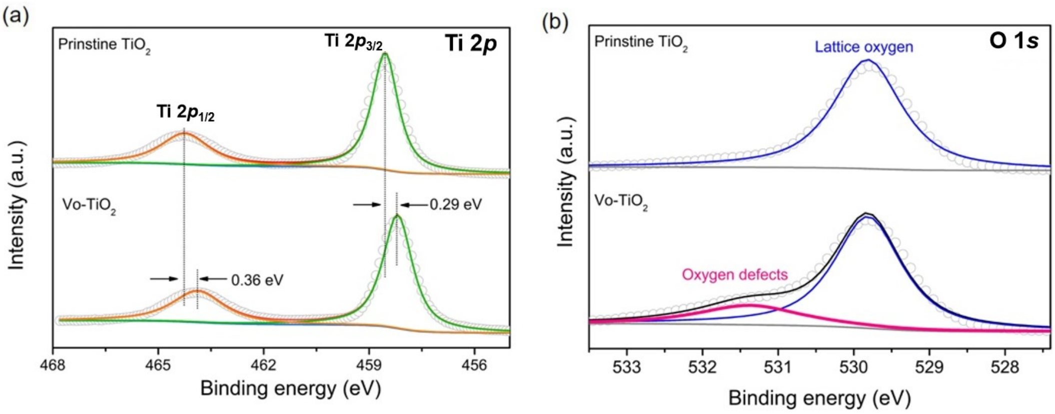

Supplementary Fig. 31 | XPS spectra of TiO_2 and Vo-TiO_2 in (a) Ti 2*p* and (b) O 1*s* regions.

The high-resolution XPS analysis of the Ti 2*p* region exhibits peaks at 458.5 and 464.3 eV, which correspond to the Ti $2p_{3/2}$ and Ti $2p_{1/2}$ states in pristine TiO_2, respectively, see Supplementary Fig. 31. Following the incorporation of V_O defects, the binding energies associated with Ti $2p_{3/2}$ and Ti $2p_{1/2}$ show negative shifts of 0.29 and 0.36 eV, respectively, indicating the presence of low-valence Ti species. Additionally, the O 1*s* spectra (Supplementary Fig. 31) reveal a notable increase in the V_O peak area for V_O-TiO_2 relative to pristine TiO_2, implying an elevated concentration of V_O defects.[30]

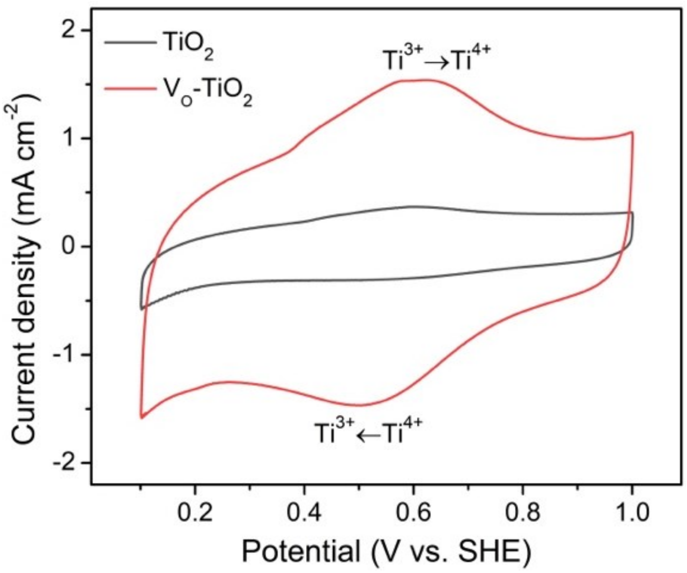

Supplementary Fig. 32 | Cyclic voltammetry tests of TiO_2 and V_O-TiO_2 systems.

We conducted cyclic voltammetry tests and observed that, compared to pristine TiO_2, V_O-TiO_2 exhibits distinct oxidation peaks corresponding to the transition from Ti^{3+} to Ti^{4+} and reduction peaks corresponding to the transition from Ti^{4+} back to Ti^{3+}, see Supplementary Fig. 32. These findings indicate that the formation of Ti^{3+} polarons in V_O-TiO_2 is potential-dependent and reversible.

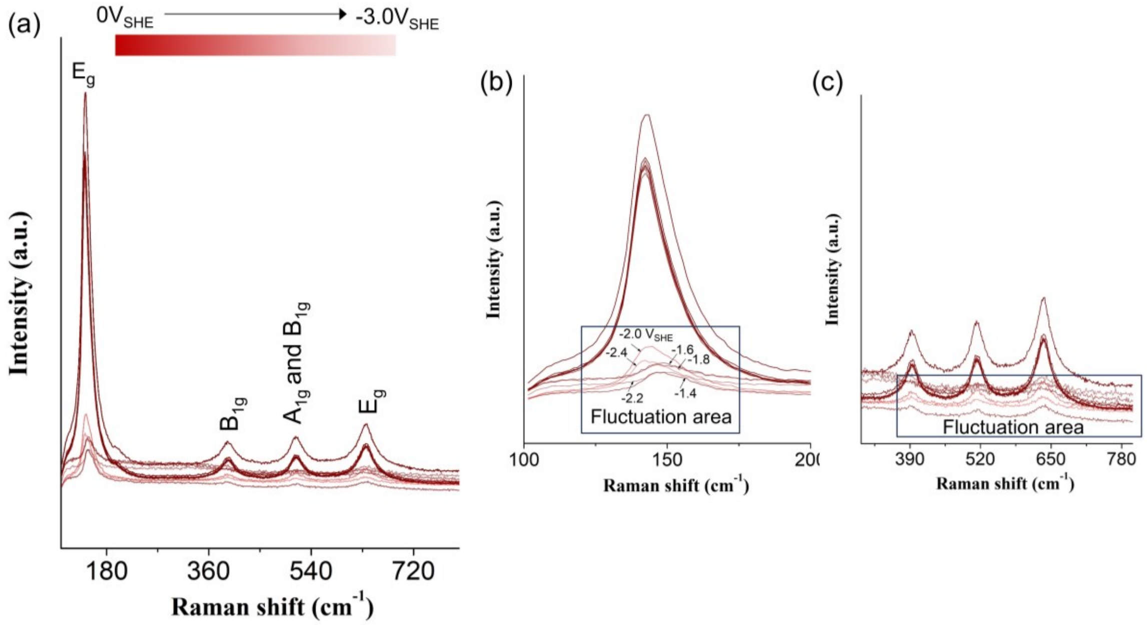

Supplementary Fig. 33 | (a) Raman spectra of TiO_2 at different electrode potentials. (b) The main Eg peak of TiO_2 at different electrode potentials. (c) The remaining three Raman peaks of TiO_2 at different electrode potentials. The main peak of pristine TiO_2 in the *in situ* Raman spectroscopies.

We analyzed all characteristic Raman modes of pristine TiO_2 at different electrode potentials. It can be clearly observed that with the application of negative potential, theE_g, B_{1g} and A_{1g} characteristic peaks exhibit significant weakening and oscillation at more negative potentials,[31,32] see Supplementary Fig. 33a. Among them, the E_g peak originates from the symmetrical bending vibration of oxygen atoms within a specific crystal plane and is the main characteristic peak of anatase; the B_{1g} peak corresponds to the asymmetrical stretching vibration of oxygen atoms along a specific crystal direction. The A_{1g} peak is related to the symmetrical stretching vibration of the Ti-O bond and reflects the degree of distortion of the octahedral [TiO_6] unit.

The *in situ* Raman spectroscopy results reveal that the peak signal of TiO_2 undergoes significant attenuation under high potential conditions, see Supplementary Fig. 33b and c. This behavior can be ascribed to the formation of oxygen vacancies or local bond length distortions within the lattice, which disrupt the symmetry of vibrational modes and consequently reduce the Raman scattering efficiency.[31,32] The intensity and frequency of A_{1g} vibration modes are related to

crystal defects, stress states and phase transitions. Therefore, through the change of the A_{1g} peak, we can find that the vibration intensity of the Ti-O bond is significantly weakened at more negative potentials (Supplementary Fig. 33c), indicating that the octahedral [TiO_6] unit undergoes distortion or an increase in lattice disorder, which may be due to the lattice distortion caused by potential-dependent Ti^{3+} polaron formation.

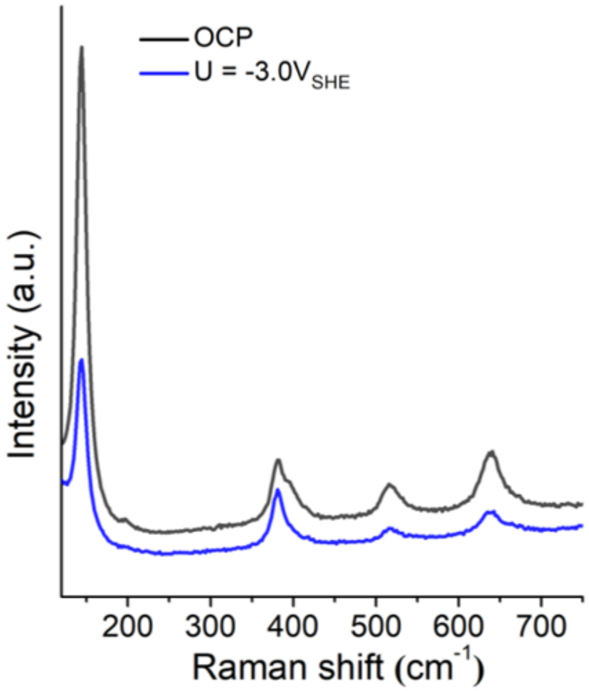

Supplementary Fig. 34 | *In situ* Raman spectroelectrochemical results of TiO_2 sample.

The *in situ* Raman results in Supplementary Fig. 34 reveal that the weakened E_g, B_{1g} and A_{1g} peaks characteristic of Ti^{3+} polaron formation present at at U = -3.0 V_{SHE} diminish under the open-circuit conditions.

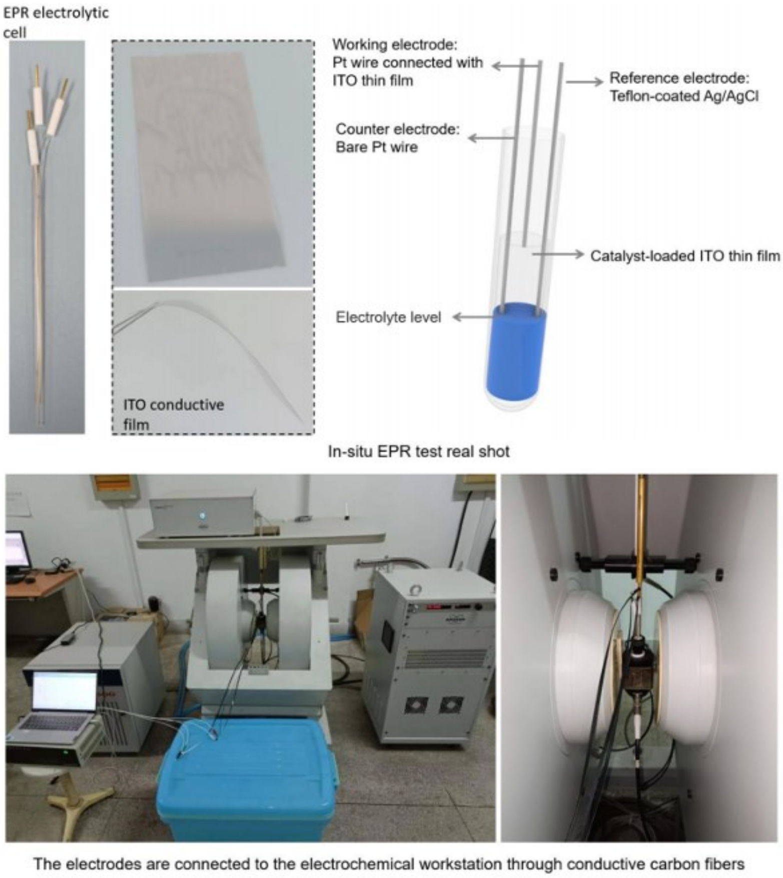

Supplementary Fig. 35 | *In situ* electrochemical EPR device design. This was originally presented in a published article from our group.[33]

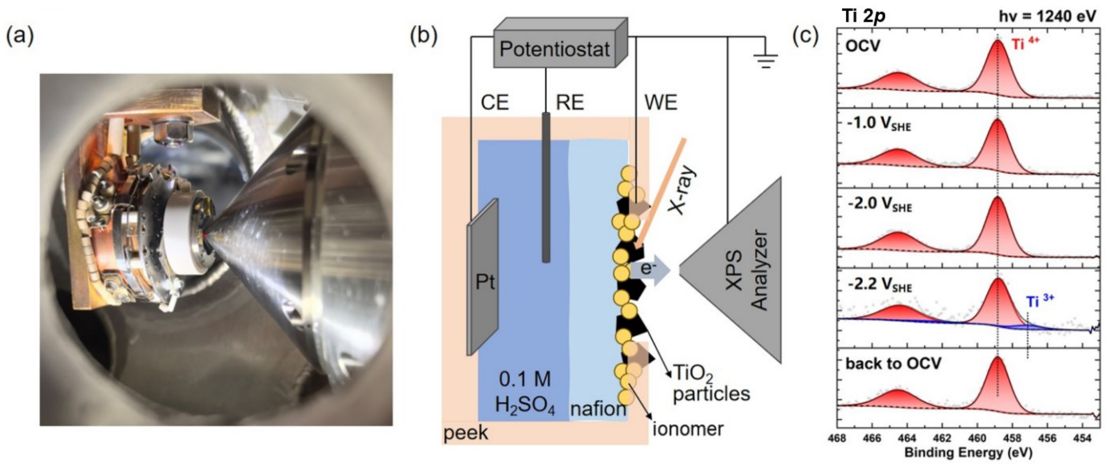

Supplementary Fig. 36 | (a) An optical image of *in situ* electrochemical cell in NAPXPS chamber. (b) A schematic of the *in situ* electrochemical measurements.

Section S8. Leveraging potential-dependent polaron formation in semiconductor electrocatalysis

Both computational and experimental results show that oxygen vacancies and polarons activate TiO_2 towards HER. Hence, one can control the HER activity by tuning the oxygen vacancy concentration through different synthesis methods and by controlling polaron formation through electrode potential. The presence of polarons depends on the presence of vacancies, and *vice versa.* Because the electrode potential controls both vacancy and polaron formation, which in turn control the overall electrocatalytic activity, electrode potential-controlled polaron or defect formation is a highly promising approach for regulating electrocatalytic activity. However, it has not received the attention it deserves. Together CIP-DFT, *in situ* electrochemical EPR, and electrocatalytic experiments clearly demonstrate that the application of an external potential directly controls the presence Ti^{3+} polarons in semiconductors, and that potential-dependent Ti^{3+} polaron formation activates TiO_2 for the HER as the thermodynamics and kinetics depend sensitively on the polaron concentration discussed above. Thus, we can generate polarons *in situ* during the reaction by controlling the reaction conditions, specifically the electrode potential, thereby enhancing HER activity. This sets our work apart from the previous studies on TiO_2 polarons generated during material preparation stages. The use of the electrode potential to control polaron formation offers several advantages over the traditional methods for defect or polaron generation:

(a) Real-time regulation and catalytic resonance theory: The electrode potential can be controlled easily to enable real-time control of polaron concentration. This allows *in situ* control over electrocatalytic activity for a large class of materials where polarons control the reactivity, including (electro)catalytically relevant metal oxides such as TiO_2, NiO, WO_3, Co_3O_4, ZnO, and CeO_2. The ability to control polaron concentration *in situ* has import implications to dynamic catalysis where the catalytic activity can be maximised through external time-dependent perturbations. The importance of this dynamic control is demonstrated by the catalytic resonance theory (CRT),[34,35] which shows that time-dependent perturbations on e.g. adsorption energy can facilitate bypassing the Sabatier scaling relations to enhance catalytic

activity. We consider the potential-dependent polaron control in reducible oxides as possible realization of CRT.

(b) Reversible modifications: Unlike doping and defect engineering, controlling the electrode potential does not result in permanent physical or chemical chances in TiO_2 material due to its high stability. This allows the system to revert to its original state if needed. Besides the CRT discussed above, the reversibility of the catalyst state is very important because under working conditions catalysts undergo structural; while chemical or structural transformations may activate the catalyst, irreversible changes in the catalyst state may also lead to loss of active site and catalysts deactiviation.[36,37] Dynamic of polaron formation is a particular realization to reversibly active an electrocatalyst.

(c) Simplified preparation: Controlling polarons through electrode potential reduces the need for complex synthesis and thereby simplifies the preparation of catalysts. In particular, the use of hazardous chemicals or high energy consumption needed for introducing structural vacancies and defects is minimized.

(d) Generality: Potential-dependent polaron formation is not only crucial for TiO_2 but we expect it also plays a significant role in other transition metal oxides; we have data to show this for NiO (Supplementary Fig. 21 and 22). Other possible materials exhibiting similar behaviour include e.g. $BiVO_4$[34] where phosphorus-doping lead to the formation of polaron states, which in turn impact the kinetics of electron transfer and water splitting, especially under higher applied potentials or hematite[27] (Fe_2O_3) in which multiple hole polarons on the electrode surface under light illumination and the application of oxidizing electrode potential significantly influences its photoelectrochemical water splitting performance.

Section S9 Overview of recent TiO_2-based HER electrocatalysts

Supplementary Table 10. Recently reported TiO_2-based electrocatalysts for HER. The blue text highlights catalysts approaching the state-of-the-art commercial Pt/C system and the green highlights state-of-the-art non-noble metal catalysts. We can clearly observe that the HER overpotentials of many TiO_2-based catalysts are already comparable to that of Pt/C. The overpotential at a current density of 10 mA/cm^2 have been widely adopted as standard measures of catalyst activity.[56]

Electrocatalyst	Electrolyte	Overpotential at j = 10 mA cm^{-2} (mV)	References	Modification strategies
Pt/C	Acidic or Alkaline	~80	42-44	
Ru/P-TiO_2	1.0 M KOH	27	38	Ru cluster on P-doped defective TiO_2
AR-TiO_2|(N,F)	1.0 M KOH	74	39	N, F co-doping-induced partial TiO_2 phase transition
Ru-TiO_2	0.1 M KOH	150	40	Ru doped-TiO_2
Ru/C-TiO_2	1.0 M KOH	44	41	Hybridizing Ru/C with amorphous TiO_2
Co-TiO_2	1.0 M KOH	78	42	Co doped-TiO_2
N/H-TiO_2	1.0 M KOH	420	43	N, H dual-doped TiO_2
H-TiO_2	1.0 M KOH	128	44	H-doped TiO_2
γ-Ti_2O_3	0.5 M H_2SO_4	271	45	Orthorhombic Ti_2O_3 polymorph
TiO_2 NDs/Co NSNTs-CFs	1.0 M KOH	40	46	Co nanotubes decorated with TiO_2 nanodots
(Co, Ni)$_2$P@TiO_2	1.0 M KOH	92	47	Defective TiO_2-coated CoNi-phosphide nanosheet

TiO_2@Ti_3C_2	1.0 M KOH	178	48	Ti_3C_2 decorated by defective TiO_2
D-TiO_2/Co@NCT	0.5 M H_2SO_4	57.5	49	Defect-rich TiO_2 nanoparticles on Co@N-doped carbon nanotubes
Co-Mo-TiO_2	1.0 M NaOH	68	50	Co-Mo-TiO_2 composite
V_O-TiO_2	1.0 M KOH	620	51	TiO_2 with oxygen vacancy
f-MWCNTs@Pd/TiO_2	PB	170	52	Pd/TiO_2 layer on functionalized multi-walled carbon nanotube
HP-TiO_2/Ni foam	1.0 M KOH	133	53	Defective TiO_2 /Ni composite
C-TiO_2	0.5 M H_2SO_4	370	54	C-doped TiO_2
$TiO_{1.23}$	1.0 M H_2SO_4	198	55	Oxygen defect-rich $TiO_{1.23}$
F-Ni_3S_2/NF	1.0 M KOH	38	56	F doped Ni_3S_2 nanosheet array
$CoSe_2$-W	1.0 M KOH	29.8	57	W doped $CoSe_2$
v-$Ni_{12}P_5$	1.0 M KOH	27.7	58	$Ni_{12}P_5$ with phosphorus vacancies

Section S10 Mechanistic insight from the Tafel slopes

Within the Butler-Volmer formalism, the HER reaction rate is given as[59]

$$k_{BV} = k_0 \exp\left(-\frac{(\gamma+\alpha)\eta F}{RT}\right) = k_0 \exp\left(-\frac{\beta\eta F}{RT}\right) \qquad (6)$$

Where γ is the number of equilibrium electron transfer steps before the rate-determining step (RDS) and α is the transfer coefficient of the RDS. k_0, η, β, F, R, and T are the intrinsic rate, over-potential, apparent transfer coefficient, Faraday constant, gas constant, and temperature, respectively.

Fig. 4b of the main manuscript shows that Tafel slope of HER on V_O-TiO_2 is close to the cardinal value of about 60 mV/dec. Within the Butler-Volmer framework this finding indicates that the 1) apparent transfer coefficient is equal to 1 (β = 1), 2) that a single electron is transferred before the transition state with highest free energy (γ= 1), and 3) that the transition state with highest free energy is related to a chemical step (α = 0) that shows no dependency on the applied electrode potential. These findings imply that HER follows the Volmer-Tafel mechanism because this is the only HER mechanism comprising a chemical (Tafel) step as the RDS:

$Volmer$: $H* + * + e^- \rightarrow 2H*$ (pre-equilibrium, γ=1)

$Tafel$: $2H* \rightarrow H_2$ (transition state with highest free energy as RDS; α= 0)

$$\beta = \gamma + \alpha \rightarrow Tafel\ slope\ of\ \frac{60mV}{dec} \qquad (7)$$

This mechanism agrees with the CIP-DFT results in the main manuscript. Despite this good agreement, it should be noted that the classical Butler-Volmer analysis contains several simplifications which may impact the analysis in our work:

- Coverage effects that may influence the rate of electrocatalytic reactions have not been fully considered. Our analysis is limited to the adsorption of two hydrogens.

- The transition-state free energy of a chemical step (Tafel step) does not depend on the applied electrode potential. We observe that the barrier of the Tafel step depends strongly on the electrode potential (Supplementary Fig. 26b)
- The formation of and charge storage in surface polarons is not described

Because the Butler-Volmer model omits the potential-dependency of polaron formation and the Tafel step, the Butler-Volmer analysis may require revision and extension for semiconductor electrodes.

References

1. Mortensen, J. J. et al. Real-space grid implementation of the projector augmented wave method. *Phys. Rev. B* **71**, 035109 (2005).
2. Enkovaara, J. et al. Electronic structure calculations with GPAW: a real-space implementation of the projector augmented-wave method. *J. Phys. Condens. Matter* **22**, 253202 (2010).
3. Wellendorff, J. et al. Density functionals for surface science: exchange-correlation model development with bayesian error estimation. *Physical Review B* **85**, 235149 (2012).
4. Korpelin, V. et al. Reducing the irreducible: dispersed metal atoms facilitate reduction of irreducible oxides. *J. Phys. Chem. C* **126**, 933-945 (2022).
5. Held, A. et al. Simplified continuum solvent model with a smooth cavity based on volumetric data. *J. Chem. Phys.* **141**, 174108 (2014).
6. Peterson, A. A. et al. Global optimization of adsorbate-surface structures while preserving molecular identity. *Top. Catal.* **57**, 40-53 (2014).
7. Larsen, A. H. et al, The atomic simulation environment—a Python library for working with atoms. *J. Phys. Condens. Matter* **29**, 273002 (2017).
8. Barmparis, G. D. et al. Thiolate adsorption on Au (hkl) and equilibrium shape of large thiolate-covered gold nanoparticles. *J. Chem. Phys.* **138**, 064702 (2013).
9. Patrick, C. E. et al. Hubbard-U corrected hamiltonians for non-self-consistent random-phase approximation total-energy calculations: a study of ZnS, TiO_2, and NiO. *Phys. Rev. B* **93**, 035133 (2016).
10. Lang, X. et al. First-principles study of configurations, electronic and photocatalytic properties of carbon-doped anatase TiO_2. *Physica B*, **624**, 413443 (2022).
11. Yu, L. et al. Oxygen reduction reaction mechanism on nitrogen-doped graphene: a density functional theory study. *J. Catal.* **282**, 183-190 (2011).
12. Melander, M. et al. Constant inner potential DFT for modelling electrochemical systems under constant potential and bias. *npj Comput. Mater.* **10**, 5 (2024).

13. Melander, M. M. Grand canonical rate theory for electrochemical and electrocatalytic systems I: general formulation and proton-coupled electron transfer reactions. *J. Electrochem. Soc*. **167**, 116518 (2020).

14. Kastlunger, G. et al. Controlled-potential simulation of elementary electrochemical reactions: proton discharge on metal surfaces. *J. Phys. Chem. C* **122**, 12771-12781 (2018).

15. Kim, D. et al. Substantial impact of charge on electrochemical reactions of two-dimensional materials. *J. Am. Chem. Soc*. **140**, 9127-9131 (2018).

16. Korpelin, V. et al. Addressing dynamics at catalytic heterogeneous interfaces with DFT-MD: Anomalous temperature distributions from commonly used thermostats. *J. Phys. Chem. Lett*. **13**, 2644-2652 (2022).

17. Islas-Vargas, C. et al. Electronic structure behavior of PbO_2, IrO_2, and SnO_2 metal oxide surfaces (110) with dissociatively adsorbed water molecules as a function of the chemical potential. *J. Chem. Phys*. **154**, 074704 (2021).

18. Liu, Y. et al. A caveat of the charge-extrapolation scheme for modeling electrochemical reactions on semiconductor surfaces: an issue induced by a discontinuous Fermi level change. Phys. *Chem. Chem. Phys*. **24**, 15511-15521 (2022).

19. Erker, S. et al. Doping dependence of the surface phase stability of polar O-terminated $(000\bar{1})$ ZnO. *New J. Phys*. **19**, 083012 (2017).

20. Campbell, Q. et al. Erratum: quantum-continuum calculation of the surface states and electrical response of silicon in solution. *Phys. Rev. B* **96**, 039901 (2017).

21. Sinai, O. et al. Multiscale approach to the electronic structure of doped semiconductor surfaces. *Phys. Rev. B* **91**, 075311 (2015).

22. Sundararaman, R. et al. Improving the accuracy of atomistic simulations of the electrochemical interface. *Chem. Rev*. **122**, 10651-10674 (2022).

23. Gonzalez, R. J. et al, Infrared reflectivity and lattice fundamentals in anatase TiO_2. *Phys. Rev*. B **55**, 7014 (1997).

24. Feng, H. et al. Activating titania for efficient electrocatalysis by vacancy engineering. *ACS Catal.* **8**, 4288 (2018).

25. He, Y. et al. Self-gating in semiconductor electrocatalysis. *Nat. Mater*. **18**, 1098-1104 (2019).

26. Yang, T. T. et al. The Bell-Evans-Polanyi relation for hydrogen evolution reaction from first-principles. *npj Comput. Mater.* **10**, 98 (2024).

27. Righi, G. et al. On the origin of multihole oxygen evolution in hematite photoanodes. *Nat. Catal*. **5**, 888-899 (2022).

28. Kwon J. et al. Oxygen vacancy creation, drift, and aggregation in TiO_2-based resistive switches at low temperature and voltage. *Adv. Funct. Mater.* ***25***, 2876-2883 (2015).

29. Wu, T. et al. Identifying the origin of Ti^{3+} activity toward enhanced electrocatalytic N_2 reduction over TiO_2 nanoparticles modulated by mixed-valent copper. *Adv. Mater*. ***32***, e2000299 (2020).

30. Dong, K. Plasma-induced defective TiO_{2-x} with oxygen vacancies: a high-active and robust bifunctional catalyst toward H_2O_2 electrosynthesis. *Chem. Catal.* ***1***, 1437-1448 (2021).

31. Zhang, H. et al. Phase engineering of MXene derivatives via molecular design for high-rate sodium-ion batteries. *Energy Environ. Mater.* **7**, e12692 (2024).

32. Vincent, M. et al. *In situ* raman spectroscopy of $Li^+$and Na^+ storage in anodic TiO_2 nanotubes: implications for battery design. *ACS Appl. Nano Mater.* **6**, 6528-6537 (2023).

33. Dong, K. *et al.* H_2O_2-mediated electrosynthesis of nitrate from air. *Nat. Synth.* **3**, 763-773 (2024).

34. Adam B. et al. Dynamic electrocatalysis: Examining resonant catalytic rate enhancement under oscillating electrochemical potential. *Chem Catal.* **2**, 3497-3516 (2022).

35. Sallye R. G. et al. Catalytic resonance theory: Negative dynamic surfaces for programmable catalysts. *Chem Catal.* **2**, 140-163 (2022).

36. Julia L. et al. Design strategies for electrocatalysts from an electrochemist's perspective, *ACS Catal.* **11**, 5318-5346 (2021).

37. Martín, A. J. et al. Unifying views on catalyst deactivation. *Nat. Catal.* **5**, 854-866 (2022).

38. Zhou, S. et al. Boosting hydrogen evolution reaction by phase engineering and phosphorus doping on Ru/P-TiO_2. *Angew. Chem. Int. Ed.* **134**, e202212196 (2022).

39. Liu, J. et al. Activating TiO_2 through the phase transition-mediated hydrogen spillover to outperform Pt for electrocatalytic pH-universal hydrogen evolution. *Small 37*, 2400783 (2024).

40. Nong, S. et al. Well-dispersed ruthenium in mesoporous crystal TiO_2 as an advanced electrocatalyst for hydrogen evolution reaction. *J. Am. Chem. Soc.* **140**, 5719-5727 (2018).

41. Wang, Y., Zhu, Q., Xie, T., et al. Promoted alkaline hydrogen evolution reaction performance of Ru/C by introducing TiO_2 nanoparticles. *ChemElectroChem* **7**, 1182-1186 (2020).

42. Li, R., Hu, B., Yu, T., et al. New TiO_2-based oxide for catalyzing alkaline hydrogen evolution reaction with noble metal-like performance. *Small Methods* **5**, 2100246 (2021).

43. Parmar, S. et al. N, H dual-doped black anatase TiO_2 thin films toward significant self-activation in electrocatalytic hydrogen evolution reaction in alkaline media. *Adv. Energ. Sust. Res.* **3**, 2100137 (2022).

44. Vadakkekara, R. et al. 3D Urchin-like Hierarchical Black TiO_2 Hollow Nanospheres: A Highly Active and Stable Electrocatalyst for Water Oxidation in Alkaline and Neutral Media. *ACS Appl. Energy Mater.* **5**, 674-684 (2021).

45. Li, Y. et al. Electronic-reconstruction-enhanced hydrogen evolution catalysis in oxide polymorphs. *Nat. Commun.* **10**, 3149 (2019).

46. Feng, J. X. et al. Efficient hydrogen evolution electrocatalysis using cobalt nanotubes decorated with titanium dioxide nanodots. *Angew. Chem. Int. Ed.* **56**, 2960-2964 (2017).

47. Liu, X. et al. Boosting electrochemical hydrogen evolution of porous metal phosphides nanosheets by coating defective TiO_2 overlayers. *Small* **42**, 1802755 (2018).

48. Deng, L., Chang, B., Shi, D., et al. MXene decorated by phosphorus-doped TiO_2 for photo-enhanced electrocatalytic hydrogen evolution reaction. *Renew. Energ.* **170**, 858-865 (2021).

49. Yu, J., Zhou, W., Xiong, T., et al. Enhanced electrocatalytic activity of Co@N-doped carbon nanotubes by ultrasmall defect-rich TiO_2 nanoparticles for hydrogen evolution reaction. *Nano Research* **10**, 2599-2609 (2017).

50. Wang, C. et al. Electrodeposited Co-Mo-TiO_2 electrocatalysts for the hydrogen evolution reaction. *J. Electrochem. Soc.* **166**, F661 (2019).

51. Feng, H. et al. Activating titania for efficient electrocatalysis by vacancy engineering. *ACS Catal.* **8**, 4288-4293 (2018).

52. Valenti, G. et al. Co-axial heterostructures integrating palladium/titanium dioxide with carbon nanotubes for efficient electrocatalytic hydrogen evolution. *Nat. Commun.* 7, 13549 (2016).

53. Yan, Y. et al. Plasma hydrogenated TiO_2/nickel foam as an efficient bifunctional electrocatalyst for overall water splitting. *ACS Sustain. Chem. Eng.* **7**, 885-894 (2018).

54. Li, Y. et al. Constructing a novel strategy for carbon-doped TiO_2 multiple-phase nanocomposites toward superior electrochemical performance for lithium ion batteries and the hydrogen evolution reaction. *J. Mater. Chem. A* **5**, 7055-7063 (2017).

55. Swaminathan, J. et al. Insights into the electrocatalytic behavior of defect-centered reduced titania ($TiO_{1.23}$). *J. Phys. Chem. C* **122**, 1670-1680 (2018).

56. He, W. et al. Fluorine-anion-modulated electron structure of nickel sulfide nanosheet arrays for alkaline hydrogen evolution. *ACS Energy Lett.* **12**, 2905-2912 (2019).

57. Zhang, J. et al. Construction of Co-Se-W at interfaces of phase-mixed cobalt selenide via spontaneous phase transition for platinum-like hydrogen evolution activity and long-term durability in alkaline and acidic media. *Adv. Mater.* **36**, 2401880 (2024).

58. Duan, J. et al. Phosphorus vacancies that boost electrocatalytic hydrogen evolution by two orders of magnitude. *Angew. Chem. Int. Ed.* **132**, 8258-8263 (2020).

59. Parsons, R. General equations for the kinetics of electrode processes, Trans. Faraday. Soc. **47**, 1332-1344 (1951)
